# Extracellular motility and cell-to-cell transmission of enterohemorrhagic *E*. *coli* is driven by EspF_U_-mediated actin assembly

**DOI:** 10.1371/journal.ppat.1006501

**Published:** 2017-08-03

**Authors:** Katrina B. Velle, Kenneth G. Campellone

**Affiliations:** Department of Molecular and Cell Biology, Institute for Systems Genomics, University of Connecticut, Storrs, Connecticut, United States of America; University of California Davis School of Medicine, UNITED STATES

## Abstract

Enteropathogenic and enterohemorrhagic *Escherichia coli* (EPEC and EHEC) are closely-related pathogens that attach tightly to intestinal epithelial cells, efface microvilli, and promote cytoskeletal rearrangements into protrusions called actin pedestals. To trigger pedestal formation, EPEC employs the tyrosine phosphorylated transmembrane receptor Tir, while EHEC relies on the multivalent scaffolding protein EspF_U_. The ability to generate these structures correlates with bacterial colonization in several animal models, but the precise function of pedestals in infection remains unclear. To address this uncertainty, we characterized the colonization properties of EPEC and EHEC during infection of polarized epithelial cells. We found that EPEC and EHEC both formed distinct bacterial communities, or “macrocolonies,” that encompassed multiple host cells. Tir and EspF_U_, as well as the host Arp2/3 complex, were all critical for the expansion of macrocolonies over time. Unexpectedly, EspF_U_ accelerated the formation of larger macrocolonies compared to EPEC Tir, as EspF_U_-mediated actin assembly drove faster bacterial motility to cell junctions, where bacteria formed a secondary pedestal on a neighboring cell and divided, allowing one of the daughters to disengage and infect the second cell. Collectively, these data reveal that EspF_U_ enhances epithelial colonization by increasing actin-based motility and promoting an efficient method of cell-to-cell transmission.

## Author summary

Enterohemorrhagic *Escherichia coli* (EHEC) and enteropathogenic *E*. *coli* (EPEC) are closely-related bacterial pathogens. EHEC infection causes bloody diarrhea and hemolytic uremic syndrome in developed nations, while EPEC is responsible for pediatric diarrhea in developing countries. Upon entry into the intestine, these pathogens form tight attachments to host cells and manipulate the cytoskeleton to create protrusions called actin pedestals. EHEC and EPEC use different mechanisms to generate actin pedestals, which are important for colonization in animal models, but it is unknown how these structures confer a colonization advantage. Our study focused on determining how hijacking the host actin cytoskeleton to form a pedestal promotes infection. We found that the EHEC pathway of pedestal formation allows for more efficient colonization of intestinal epithelial cells than the EPEC pathway. Additionally, we directly show how actin pedestals allow the bacteria to spread from one infected cell to a neighboring healthy cell. These findings advance our understanding of the molecular and cellular basis of EPEC and EHEC colonization, which is important for developing new ways to combat infections with these bacteria.

## Introduction

Many pathogens reorganize the cytoskeleton of their host cells during the course of infection. These include the intracellular bacteria *Listeria monocytogenes* and *Shigella flexneri*, which generate filamentous actin “comet tails” that propel the bacteria through the cytosol, drive the formation of membrane protrusions, and ultimately spread the infection to neighboring cells [1–3]. Enterohemorrhagic and enteropathogenic *Escherichia coli* (EHEC and EPEC) are also capable of reorganizing actin, but these pathogens remain extracellular and signal across the plasma membrane to create structures called attaching and effacing (A/E) lesions [4]. A/E lesions are characterized by intimate attachment of the bacteria to the host cell membrane, a loss of microvilli, and assembly of filamentous actin “pedestals” beneath the bacteria [5, 6]. The ability to form these lesions correlates with pathogenesis, because EHEC and EPEC mutants that are unable to adhere intimately to host cells fail to colonize or cause disease in animal models [7–11], intestinal explants [12], and human volunteers [13, 14]. Since the discovery of pedestals nearly three decades ago [6], the mechanisms of actin assembly within these structures have been fairly well characterized. However, the function of pedestals in the cellular basis of disease remains relatively unclear.

To generate actin pedestals, EHEC and EPEC each use a type three secretion system (T3SS) for injecting effector proteins into mammalian host cells [15]. Among the numerous effectors is the translocated intimin receptor, Tir, which is inserted into the plasma membrane and binds to the adhesin intimin expressed on the bacterial surface, thereby forming a tight attachment to the host cell [16, 17]. The EPEC version of Tir becomes tyrosine phosphorylated at residue 474 by host cell kinases [18–20] and binds to the adaptor proteins Nck1 and Nck2 [21, 22], which recruit N-WASP [23], an activator of the Arp2/3 complex that promotes actin nucleation to form a pedestal [24]. The EHEC version of Tir does not become tyrosine phosphorylated [17], but recruits the host proteins IRTKS [25] and IRSp53 [26] which interact with the EHEC effector protein EspF_U_, a multivalent and potent activator of N-WASP [27–31]. Although EPEC and EHEC pedestals are triggered by different signaling mechanisms, they are morphologically indistinguishable and contain many of the same host factors [32, 33], leading to the widespread belief that their pathogenic functions are similar or equivalent [33].

Early studies suggested that intimate attachment to the plasma membrane by EPEC may play a role in evading phagocytosis [34] or that the intimin-Tir-actin interactions function to anchor the bacteria to the host cell by linking them to the cytoskeleton [35]. However, additional work demonstrated that actin pedestals are dynamic and drive a form of actin-based motility that allows the bacteria to “surf” on top of cultured cells [36, 37]. More recent studies implied that actin assembly enhances effector entry either directly or indirectly [38, 39], while another has indicated that Tir tyrosine signaling is important for colonization *in vivo* [40]. Furthermore, actin pedestals appear to promote more stable attachments to cultured cells or to the intestinal mucosa in animal models [39, 41, 42]. For example, an EHEC strain capable of intimate adherence but deficient in EspF_U_-mediated actin pedestal assembly was less abundant in the intestines of experimentally infected infant rabbits and gnotobiotic piglets than wild type EHEC [42]. Most strikingly, the use of *Citrobacter rodentium* strains to model EPEC/EHEC infections revealed that intestinal N-WASP knock-out mice were resistant to infection, and that bacteria harboring a tyrosine-to-phenylalanine Tir mutant colonized the colon in wild type mice less efficiently [41]. Despite this progress, the precise cellular basis for how pedestals promote or enhance colonization has yet to be clearly defined.

The current study focused on characterizing the roles of pedestals in anti-phagocytosis, bacterial motility, and epithelial cell colonization. Our findings indicate that EHEC and EPEC pedestals serve similar functions with respect to resisting phagocytosis by macrophages and enabling actin-based motility on non-polarized cells. However, on polarized epithelial cells, EHEC pedestals confer a colonization advantage, allowing for the formation of large “macrocolonies” that encompass several host cells. This EHEC-specific advantage stems from faster EspF_U_-driven motility and a previously unrecognized mechanism of cell-to-cell bacterial transfer.

## Results

### EPEC and EHEC pedestals can be compared directly using engineered EPEC strains

**Fig 1:**
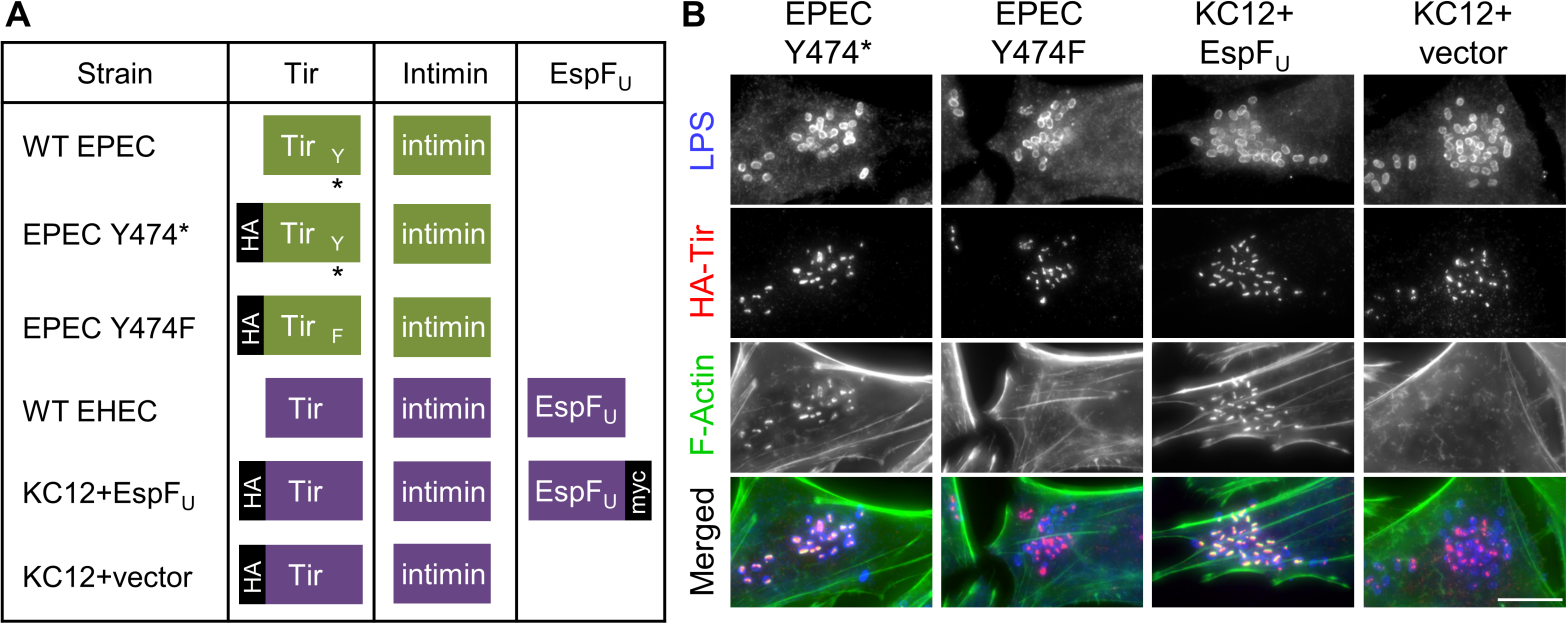
EHEC and EPEC pedestals can be compared directly using engineered EPEC strains. (A) EPEC Y474*, EPEC Y474F, KC12+EspF_U_, and KC12+vector are all EPEC strains engineered to express HA-tagged versions of Tir and/or myc-tagged EspF_U_ to reflect the WT EPEC or WT EHEC pathways of pedestal assembly. Green and purple boxes represent EPEC and EHEC proteins, respectively. The asterisk indicates phosphotyrosine residue 474. (B) HeLa cells were infected for 3 h with the indicated strains, fixed, and stained to visualize LPS, HA-Tir, and F-actin. Scale bar, 10 μm.

EPEC is able to infect cultured cells better than EHEC *in vitro* [43, 44], and EHEC has a more extensive repertoire of effector proteins than EPEC [15]. Therefore, to study phenotypic differences stemming specifically from the divergent pedestal assembly pathways, we used EPEC strains engineered to express epitope-tagged versions of Tir or EspF_U_ that generate pedestals by either the EPEC or EHEC mechanism. Actin pedestal assembly driven by EPEC Tir relies heavily upon the phosphorylation of tyrosine 474, so to enable immunostaining of EPEC Tir variants capable or incapable of Y474 phosphorylation, we employed two strains of EPEC engineered with chromosomal deletions of *tir* that harbor low copy number plasmids encoding HA-tagged wild type Tir or a Y474F point mutant [22]. In agreement with previous results showing that Y474 is required for >95% of actin pedestal formation [21, 45, 46], these two strains, referred to as EPEC Y474* and EPEC Y474F (Fig 1A), were each capable of translocating HA-Tir into HeLa cells, but only EPEC Y474* triggered actin polymerization into intensely-staining pedestals (Fig 1B).

In contrast to EPEC, the EHEC pathway of actin polymerization is largely dependent on clustering of the multivalent effector protein, EspF_U_ [31]. To directly compare bacteria proficient for the EspF_U_-driven mechanism of pedestal assembly to bacteria utilizing EPEC Tir, we employed an EPEC strain encoding HA-tagged EHEC Tir and EHEC intimin in place of the endogenous chromosomal copies of the EPEC genes for Tir and intimin [22]. Referred to as KC12, this strain was transformed with a low copy number plasmid encoding myc-tagged EspF_U_ (KC12+EspF_U_) to enable pedestal formation (Fig 1A). KC12 harboring an empty vector (KC12+vector) was used as a pedestal-deficient control strain [27]. As expected, both KC12 derivatives translocated HA-Tir into HeLa cells, but intense actin pedestals were only assembled by the EspF_U_-expressing bacteria (Fig 1B). These strains, isogenic with the exception of the pedestal effectors, were well suited to characterize the functions of the different actin assembly pathways on multiple cell types.

### The EPEC and EHEC mechanisms of pedestal assembly both provide anti-phagocytic functions

Intestinal macrophages serve as an initial line of innate defense in the gut, as they phagocytose bacteria and help maintain tissue homeostasis [47]. Macrophages are also known to rely on N-WASP and the Arp2/3 complex to form protrusions used to engulf bacteria [48]. To test whether the direct manipulation of the actin nucleation machinery by EHEC or EPEC might prevent phagocytosis, human THP-1 monocytes were activated to a macrophage-like state and infected with the strains of KC12 or EPEC that could or could not form pedestals. KC12+EspF_U_ and EPEC Y474* both retained the capacity to translocate Tir and form pedestals on this cell type, while KC12+vector and EPEC Y474F were only capable of Tir translocation (S1A Fig). To differentiate external bacteria from total bacteria, outside-inside staining was performed. These experiments revealed that some bacteria were internalized by THP-1 cells (S1B Fig). LAMP-1 staining of cells infected in parallel confirmed that internalized bacteria were associated with lysosomes (S1C Fig), suggesting that the intracellular bacteria were indeed phagocytosed and targeted for degradation.

To determine if there was a discernable difference in phagocytosis of the KC12 and EPEC strains, differential outside-inside staining of infected cells was used to quantify the proportions of extracellular versus intracellular bacteria at various time points (S1D Fig). Extracellular KC12+EspF_U_ and EPEC Y474* were both found in greater quantities than their pedestal deficient counterparts between 30 and 150 min post infection (S1D Fig). Despite recruiting the actin assembly machinery by different mechanisms, resistance to internalization by KC12+EspF_U_ and EPEC Y474* was nearly identical, as approximately 75% of each strain remained extracellular at 90 min. In contrast, the pedestal-deficient strains KC12+vector and EPEC Y474F were internalized in amounts equivalent to a strain that completely lacked the type 3 secretion system (EPECΔT3SS) (S1D Fig). Thus, the ability to form pedestals by either the EHEC or EPEC pathway of actin assembly allows for increased resistance to phagocytosis.

### Both mechanisms of actin pedestal assembly promote motility and exploration of the cell surface

**Fig 2:**
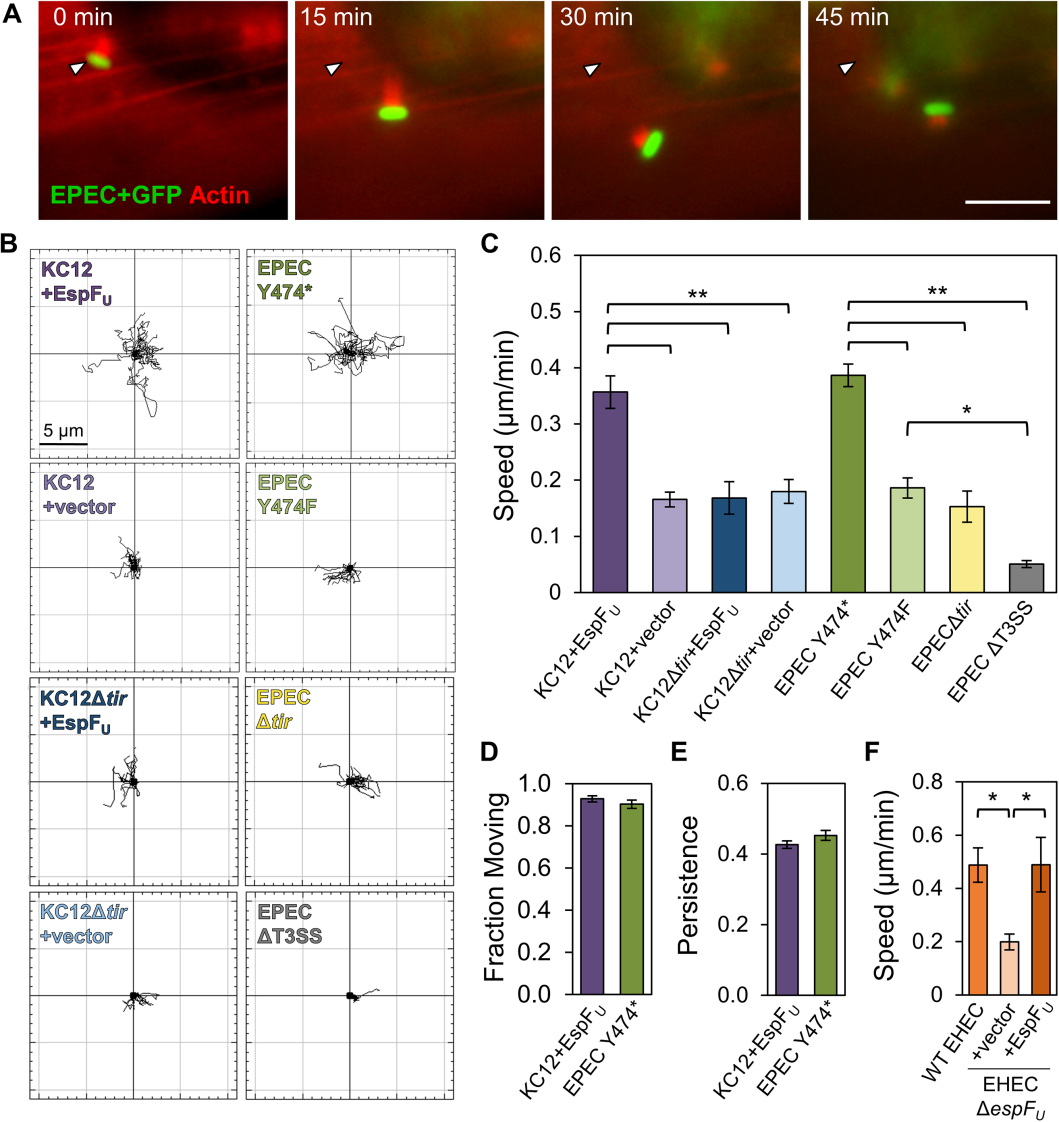
The actin pedestals assembled by KC12+EspF_U_ and EPEC Y474* promote motility and exploration of the host cell surface. (A) NIH3T3 cells stably expressing mCherry-actin were infected with EPEC expressing GFP for 3 h and imaged live for 45 min. Scale bar, 10 μm. (B) mCherry-actin expressing cells were infected with the indicated strains for 3 h and imaged live for 18–20 min. 15–20 bacteria per host cell for each strain were tracked, and data from representative cells were plotted such that points starting at t = 0 were centered at the origin. (C) Bacterial motility rates were quantified from cells infected as in (B). Each bar represents the mean speed (+/- SE) of bacteria on 6–20 host cells (95–293 total bacteria). ** p<0.01, *p<0.05 (ANOVA, Tukey post-hoc tests). (D) The fraction of pedestals that were considered moving was quantified, using the average speed of the pedestal deficient counterpart strain as a minimum cutoff to define movement. Each bar represents the mean (+/- SE) from 227–320 pedestals. p = 0.3 (Fisher’s exact test). (E) Directional persistence of pedestals was calculated by dividing the maximum displacement by the total path length for pedestals considered to be moving. Each bar represents the mean (+/- SE) for 205–297 pedestals on 19–20 cells. p = 0.1 (unpaired *t* test) (F) Cells were infected with EHEC strains with or without EspF_U_ and imaged live. Each bar represents the mean speed (+/- SE) of bacteria on 6 cells (60 bacteria). *p<0.05 (ANOVA, Tukey post-hoc test).

EPEC and EHEC actin pedestals were shown to enable bacterial movement, or “surfing” on host cells many years ago, and EPEC was initially found to move at a maximum speed of 4.2 μm/min [36]. A follow-up study determined that actin polymerization rates within pedestals ranged between 0.2 and 1.0 μm/min [37]. However, aside from those preliminary measurements, the properties of EPEC and EHEC movement have not been directly compared. To explore the similarities and differences in motility among bacteria forming EPEC Tir-dependent pedestals versus EspF_U_-dependent pedestals, NIH3T3 fibroblasts stably expressing mCherry-actin were infected with EPEC or KC12 derivatives, and examined live (Fig 2A, S1 Video). The general movements of actin pedestals that we observed were consistent with previous descriptions [36], as some pedestals remained fairly stationary, while others translocated along the cell surface (Fig 2A).

We next tracked the movements of individual bacteria and plotted them so that the starting point at time = 0 was placed at the graph origin (Fig 2B). These experiments illustrated that pedestal proficient bacteria explored the cell surface to a greater extent than pedestal deficient strains, with KC12+EspF_U_ and EPEC Y474* typically traveling through areas with radii of 6–7 μm over an 18 min period, compared to radii of <4 μm for the pedestal deficient KC12+vector and EPEC Y474F bacteria (Fig 2B). Measurements of the speeds of each strain further demonstrated that KC12+EspF_U_ and EPEC Y474* moved at very similar average rates (0.36 and 0.39 μm/min, respectively) and maximum rates (4.14 and 4.20 μm/min, respectively), and were each twice as fast as their pedestal deficient counterparts KC12+vector and EPEC Y474F (average rates of 0.17 and 0.19 μm/min, respectively), whose limited movements could be largely attributed to the fluidity of the plasma membrane or host cell stretching (Fig 2C). Additionally, KC12 and EPEC derivatives completely lacking Tir showed restricted movements similar to those of KC12+vector and EPEC Y474F, (Fig 2B and 2C), indicating that intimate adherence by the latter strains in the absence of a pedestal does not provide a measurable motility advantage. However, strains lacking Tir were still faster than the T3SS mutant, suggesting that the T3SS mutant did not interact with the plasma membrane extensively enough to be influenced by the movement of the underlying cell. (Fig 2B and 2C).

To assess potential motility differences between the pedestals of KC12+EspF_U_ and EPEC Y474*, live imaging data was subjected to further quantitative analyses. Based on pedestal tracking, and using the average speeds of pedestal deficient bacteria as background values for defining movement, the fraction of pedestals classified as moving was calculated. By these criteria, equivalent fractions of KC12+EspF_U_ and EPEC Y474* pedestals were scored as moving (Fig 2D), indicating that both the EPEC Tir-dependent and the EspF_U_-dependent mechanism of actin polymerization promote similar frequencies of motility. Additionally, directional persistence was measured by dividing the displacement of each pedestal by the total path length, making a value of 1 a directly linear route. Both sets of pedestals had values just over 0.4, indicating that KC12+EspF_U_ and EPEC Y474* display comparable degrees of directional persistence (Fig 2E). Finally, to ensure that the same trend of pedestal-associated motility that we observed for KC12+EspF_U_ was exhibited by *bona fide* EHEC, movements of WT EHEC, as well as EHECΔ*espF*_*U*_ mutant strains harboring either the EspF_U_-myc plasmid or an empty vector control were examined. As expected, the presence of EspF_U_ allowed for significantly faster motility than the pedestal deficient strain (Fig 2F). Collectively, these data highlight numerous similarities between the EHEC and EPEC pedestal assembly pathways in motility and exploration of the surface of non-polarized cells.

### KC12 and EPEC strains form “macrocolonies” that grow over time on polarized epithelial cells

**Fig 3:**
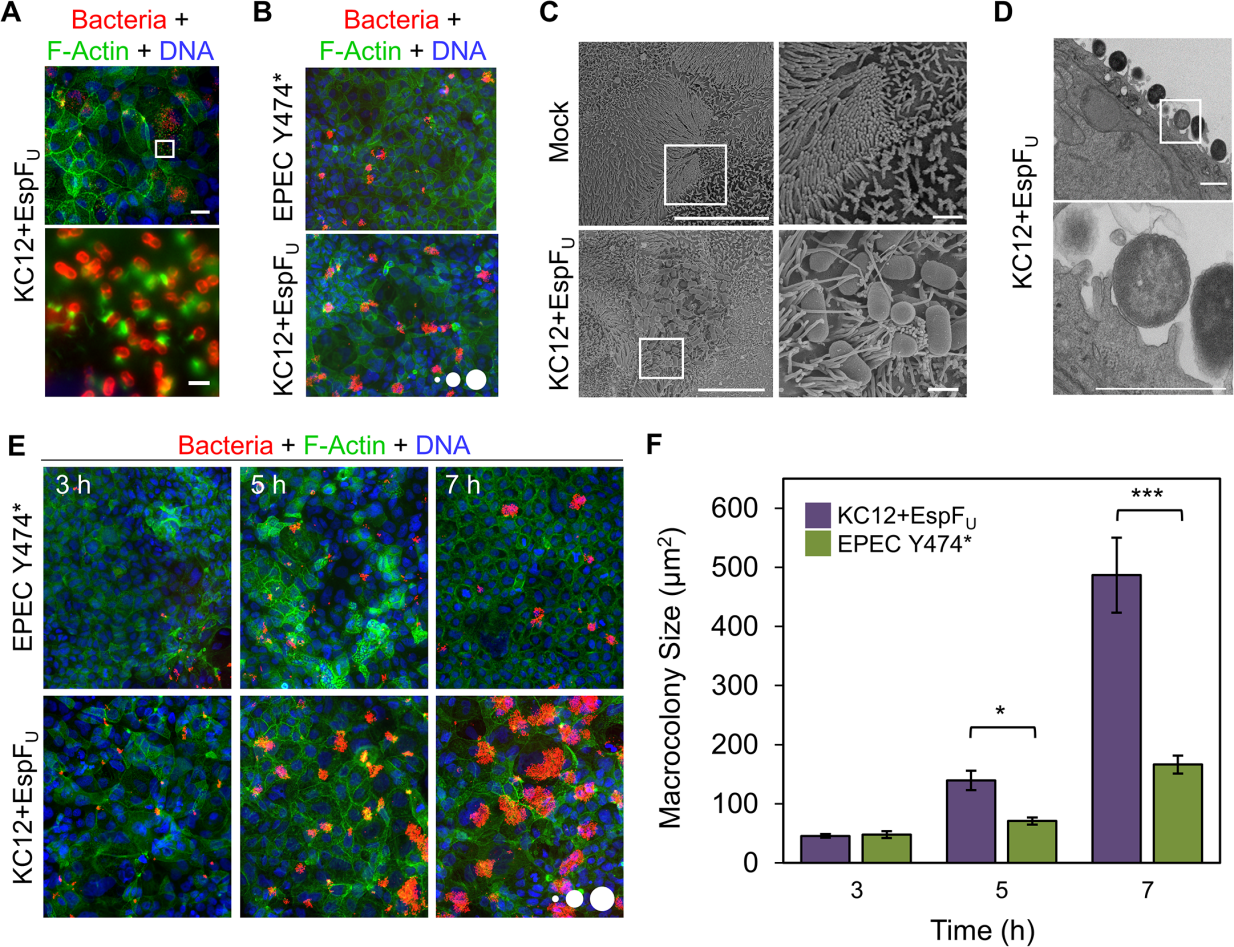
KC12+EspF_U_ and EPEC Y474* form macrocolonies that grow over time on polarized epithelial cells. (A) Polarized Caco-2 monolayers were infected with KC12+EspF_U_ for 6 h, fixed, and stained for LPS, DNA, and F-actin. Scale bar, 25 μm; inset 2.5 μm. (B) Cells infected in (A) with KC12+EspF_U_ or EPEC Y474* were imaged at a lower magnification. Scale circles have areas of 100, 500, and 1000 μm^2^. (C) Polarized Caco-2 monolayers were mock infected (top panels) or infected for 6 h with KC12+EspF_U_ (bottom panels), and visualized by scanning electron microscopy. The inset highlights a portion of a macrocolony. Scale bars, 10 μm; inset,1 μm. (D) Cells infected in parallel with those in (C) were visualized by transmission electron microscopy. The inset shows a cross-section of a pedestal. Scale bars, 2 μm. (E) Polarized Caco-2 monolayers were infected for 3, 5, or 7 h, fixed, and stained to visualize bacteria, DNA, and F-actin. Scale circles, 100, 500, 1000 μm^2^. (F) Macrocolony sizes were quantified from cells infected as in (E). Each bar represents the mean (+/- SE) of macrocolony sizes calculated from 3–6 coverslips (85–2309 colonies). Macrocolonies over 25 μm^2^ were included in quantification. *p<0.05, ***p<0.001 (unpaired *t* tests).

While our macrophage and fibroblast infections revealed many parallels in function between EspF_U_- and phospho-Tir-mediated pedestals on these cell types, the ultimate target host cells for EPEC and EHEC are polarized intestinal epithelial cells. To explore potential pedestal-related similarities and differences on epithelial monolayers, we infected polarized JEG-3 (human placental epithelial) and Caco-2 (human colonic epithelial) cells. Staining with antibodies to Ezrin and ZO-1 confirmed that, in contrast to nonpolarized HeLa epithelial cells, JEG-3 and Caco-2 cells contained microvilli and formed tight junctions (S2A Fig). As expected, 6 h of infection resulted in a loss of microvilli, especially in cells with high bacterial burdens (S2B Fig). However, ZO-1 staining was not extensively disrupted at this time point (S2A Fig), and disruption did not correlate with bacterial load (S2B Fig). Immunostaining of adherent bacteria and imaging at high magnification confirmed the presence of actin pedestals (Fig 3A).

Interestingly, imaging at low magnification revealed an unexpected colonization phenotype. KC12+EspF_U_ and EPEC Y474* formed discrete infection foci, or “macrocolonies,” on the polarized cell monolayers (Fig 3B). These were reminiscent of colonies formed on an agar plate or plaques generated by intracellular pathogens. We chose the term macrocolonies to distinguish these structures from the definition of microcolonies as multiple loci of infection on a single cell, or localized adherence patterns driven by bundle-forming pili [49, 50], because they were larger and spanned several host cells.

To obtain a higher resolution view of KC12+EspF_U_ macrocolonies, electron microscopy was conducted on uninfected and infected polarized Caco-2 cells. Scanning electron micrographs confirmed the immunostaining results, in that the uninfected cells were extensively microvilliated. But, after infection, patches lacking microvilli became apparent while some microvilli had coalesced towards bacteria (Fig 3C), as demonstrated previously for EPEC infections [51]. Similar to our fluorescence microscopy results, bacteria were found in distinct macrocolonies on the monolayer (Fig 3C). Transmission electron microscopy of samples prepared in parallel allowed for sectioning through a macrocolony, and revealed that bacteria generated pedestals (Fig 3D). Collectively, these findings indicate that EPEC and KC12 strains colonize polarized monolayers by forming discrete infection foci, or macrocolonies, which encompass multiple host cells.

To further characterize macrocolony biogenesis on Caco-2 cells, we fixed cells at various time points after infection (Fig 3E). At 3 h, colonies were small and were often restricted to one host cell. By 5 h, colonies had increased in size, and a striking difference in colony growth between strains began to emerge, with KC12+EspF_U_ macrocolonies growing significantly larger than EPEC Y474* macrocolonies (Fig 3F). By 7 h, this difference was exacerbated, as the average size of KC12+EspF_U_ colonies was nearly three-fold larger than those formed by EPEC Y474* (Fig 3E and 3F). Although macrocolonies of both strains grew over time, the faster expansion kinetics exhibited by KC12+EspF_U_ highlights a significant and unexpected colonization difference arising from the two different pedestal assembly pathways.

### EHEC Tir and EspF_U_ promote more efficient colonization of polarized epithelial cells than EPEC Tir

**Fig 4:**
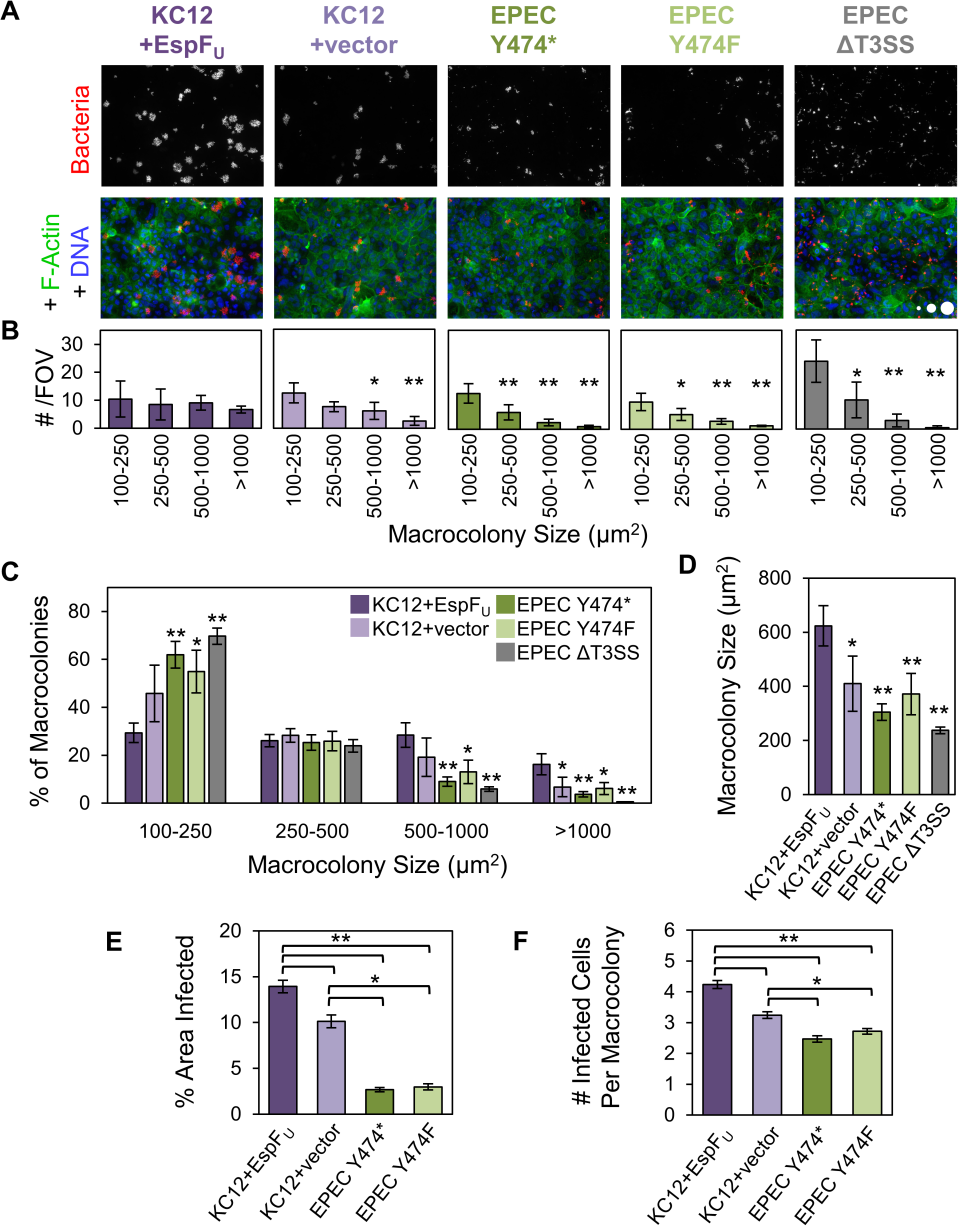
EHEC Tir and EspF_U_ promote more efficient colonization of polarized epithelial cells than EPEC Tir. (A) Polarized Caco-2 monolayers were infected with KC12 and EPEC strains for 6 h, fixed, and stained to visualize bacteria, F-actin, and DNA. Scale circles, 100, 500, 1000 μm^2^. (B) Macrocolony sizes >100 μm^2^ from experiments described in (A) were measured and binned into size groups. Each bar represents the mean number of macrocolonies (+/- SE) from 3 experiments, spanning 225–275 fields of view (FOV) and 4658–8434 colonies. All p-value significance is in reference to the 100–250 μm^2^ bins. *p<0.05, **p<0.01 (ANOVA, Tukey post-hoc tests). (C) Data collected in (B) were reorganized to compare the strains within each category. Bars represent the mean (+/- SD) for 3 experiments, of the % of colonies falling into each bin. Asterisks are in reference to KC12+EspF_U_. *p<0.05, **p<0.01 (ANOVA, Tukey post-hoc tests). (D) Macrocolony sizes measured from part (B) were averaged. Each bar represents the mean (+/- SD) from 3 experiments. All p-values are in reference to KC12+EspF_U_. (E) Experiments were performed as in (A), but for 7 h. Each bar represents the mean (+/- SE) of the % of monolayer area infected for 58–60 FOVs. (F) The number of infected cells per macrocolony was calculated. Each bar represents the mean (+/- SE) calculated from 238–497 macrocolonies, taken from 17–19 representative fields of view from the images quantified in (E). *p<0.05, **p<0.01 (ANOVA, Tukey post-hoc tests).

To further investigate the difference in macrocolony size between KC12+EspF_U_ and EPEC Y474*, polarized Caco-2 monolayers were infected with these strains, and subjected to frequent washes and media changes to remove unbound bacteria and promote the development of macrocolonies exclusively from bacteria that adhered within the first hour of infection. After 6 h, monolayers were fixed and analyzed by fluorescence microscopy, and the area of each macrocolony over 100 μm^2^ was measured. In agreement with the time course data, quantification and histogram analysis revealed that KC12+EspF_U_ colonies were notably larger than EPEC Y474* colonies (Fig 4A–4D). This difference was especially apparent when considering structures over 1000 μm^2^, because these made up 16% of all colonies for KC12+EspF_U_, but <4% of those for EPEC Y474* (Fig 4B and 4C). After 7 h of infection, this difference in colony size translated to a larger infected area of the monolayer (Fig 4E), and a greater number of infected cells per macrocolony (Fig 4F), despite indistinguishable bacterial multiplication rates under these culture conditions (S3 Fig).

To examine if pedestals play a role in determining macrocolony size, cells were also infected with KC12+vector and EPEC Y474F. KC12+vector macrocolonies were significantly smaller than those of their pedestal proficient counterpart (Fig 4A–4D), and while KC12+EspF_U_ had approximately equal numbers of macrocolonies of all size ranges, KC12+vector had many more colonies in the small 100–250 μm^2^ range than in categories above 500 μm^2^ (Fig 4A and 4B). Furthermore, KC12+vector infected a smaller percentage of the monolayer, and fewer host cells per macrocolony (Fig 4E and 4F), indicating that EspF_U_ enhances colony size. In contrast, EPEC Y474* did not have any colony size advantage over EPEC Y474F. The size distributions for these two strains were very similar (Fig 4B), and there was no significant difference in average size, area infected, or the number of cells infected per macrocolony (Fig 4D–4F). Surprisingly, KC12+vector infected a larger area of the monolayer and more cells per colony than any of the EPEC Tir-expressing strains, suggesting an important role for the EHEC version of Tir in enhancing macrocolony size. As expected, the T3SS mutant yielded very petite colonies, with 70% falling into the small 100–250 μm^2^ range (Fig 4A–4C). Collectively, these data suggest that the EHEC mechanism of pedestal assembly provides a colonization advantage over the EPEC pathway of actin polymerization in infections of polarized intestinal epithelia.

### EHEC Tir, EspF_U_, and the host Arp2/3 complex drive KC12 macrocolony expansion

**Fig 5:**
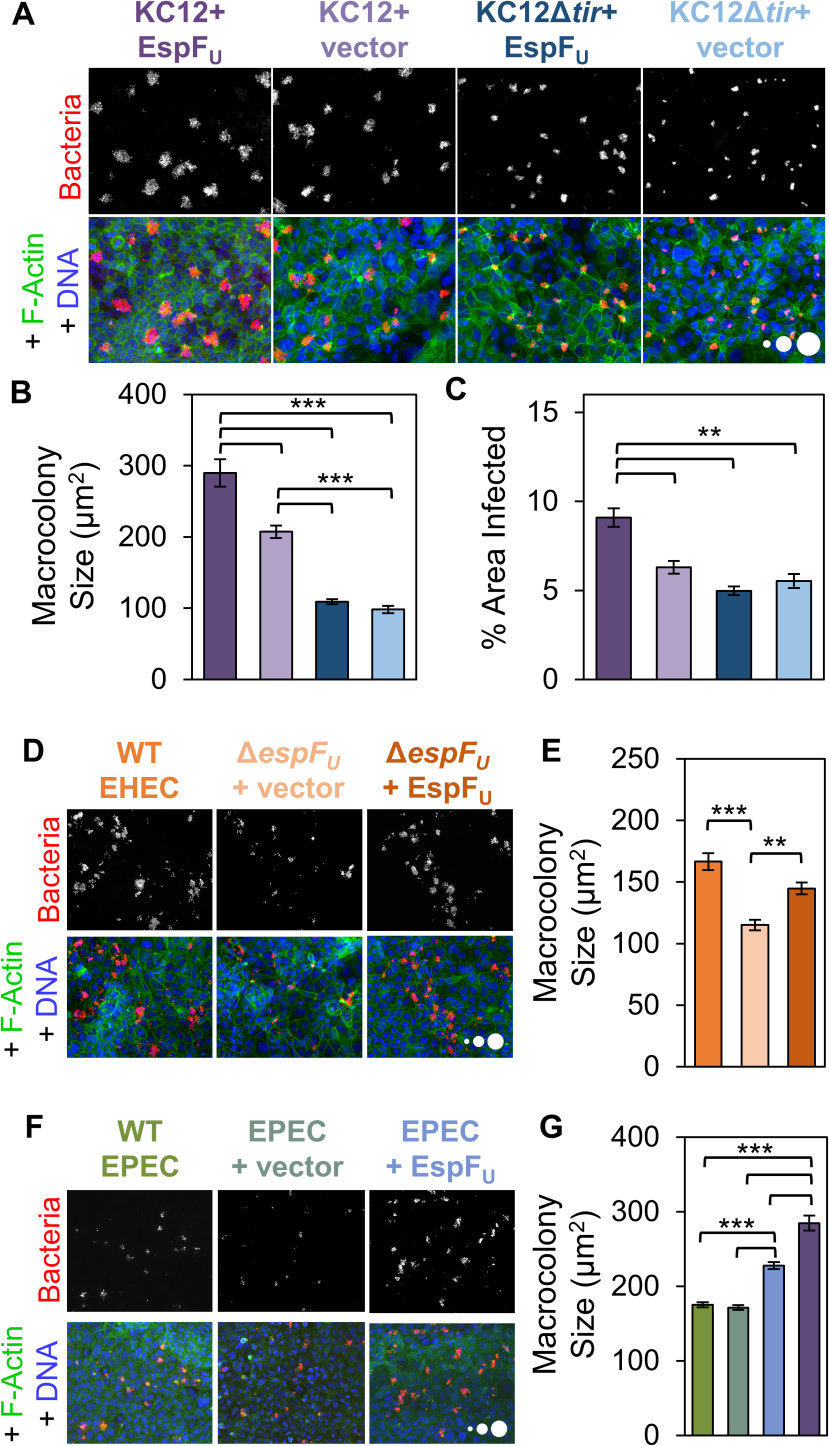
EspF_U_ can enhance macrocolony size using either the EHEC or EPEC version of Tir. (A) Polarized Caco-2 monolayers were infected for 6 h with the indicated KC12 and KC12Δ*tir* strains, fixed, and stained to visualize bacteria, DNA, and F-actin. (B) Experiments described in (A) were quantified. Each bar represents the mean macrocolony size (+/- SE) calculated from 6 coverslips (2025–3179 colonies). (C) The experiments in (B) were also used to quantify the % of monolayer area infected. Each bar represents the mean (+/- SE) from 59–60 FOVs. (D-E) Polarized Caco-2 monolayers were infected with EHEC strains for 8 h, fixed, and stained as in (A). Bars represent the mean macrocolony size (+/- SE) calculated from 315–617 macrocolonies. (F-G) Polarized Caco-2 monolayers were infected with WT EPEC strains with or without EspF_U_. Each bar represents the mean macrocolony size (+/- SE) calculated from 1163–2722 macrocolonies. KC12+EspF_U_ is shown in purple. For all panels, scale circles, 100, 500, 1000 μm^2^. ** p <0.01, *** p <0.001 (ANOVA, Tukey post-hoc tests). To allow for a sufficient number of Δ*tir* colonies that could be analyzed, colonies larger than 50 μm^2^ were included in quantification, unlike Fig 4 where 100 μm^2^ was the lower limit.

To further characterize the role of EHEC Tir in colonization, KC12 and KC12Δ*tir* strains harboring EspF_U_-myc or control plasmids were used to infect Caco-2 monolayers. These variants allowed for an assessment of the individual and combined contributions of Tir and EspF_U_ to colonization. Again, KC12+EspF_U_ formed larger macrocolonies and infected a greater fraction of the epithelial tissue than KC12+vector (Fig 5A–5C). In addition, both strains with *tir* deletions formed smaller colonies than the Tir-expressing strains (Fig 5A and 5B). EspF_U_ was unable to rescue this defect of KC12Δ*tir* (Fig 5A and 5B), suggesting that EspF_U_ requires Tir to contribute to colonization. Collectively, these results show that EHEC Tir can promote the development of macrocolonies and that its effects are enhanced by EspF_U_.

To ensure that EspF_U_ plays an equally important role in colonization by EHEC, Caco-2 monolayers were infected with WT EHEC, as well as an EspF_U_ deletion strain and a complemented strain expressing EspF_U_-myc. Macrocolonies of EHECΔ*espF*_*U*_+vector were significantly smaller than WT EHEC, and this deficiency was rescued by the EspF_U_-myc plasmid (Fig 5D–5E). Since EspF_U_ is capable of interacting with the EPEC version of Tir to promote Nck-independent actin polymerization [52], we further hypothesized that adding EspF_U_ to EPEC might enhance EPEC macrocolony size. In fact, infection of polarized Caco-2 monolayers with EPEC+EspF_U_ confirmed that EspF_U_ localized to EPEC pedestals (S4A Fig), and revealed that EPEC+EspF_U_ macrocolonies were 25% larger than those formed by WT EPEC (Fig 5F–5G). However, KC12+EspF_U_ still produced the largest macrocolonies, underscoring the importance of EHEC Tir (Fig 5G). These data indicate that EspF_U_ is able to enhance macrocolony size using either the EHEC or EPEC version of Tir.

**Fig 6:**
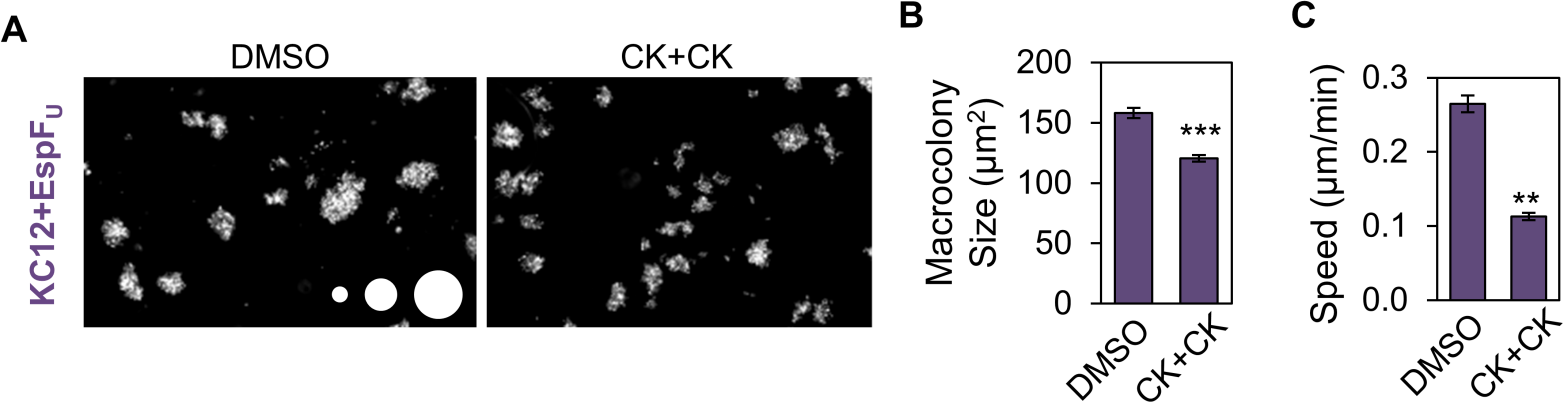
The Arp2/3 complex is important for macrocolony size and actin-based motility. (A) Polarized Caco-2 monolayers were treated with DMSO or the Arp2/3 inhibitors CK666+CK869 (CK+CK), infected with KC12+EspF_U,_ fixed, and stained for bacteria. Scale circles, 100, 500, 1000 μm^2^. (B) The average colony size was quantified from experiments performed in (A). Each bar represents the mean (+/- SE) calculated from 640–706 colonies spanning 4–6 coverslips from 3 experiments. Colonies larger than 25 μm^2^ were included in analysis. *** p<0.001 (unpaired *t* test). (C) NIH3T3 cells expressing mCherry-actin were infected with KC12+EspF_U_ and treated with DMSO or CK666+CK869. Mean speeds (+/- SE) were calculated based on pedestals from 13–15 cells per condition from at least 3 experiments (185–220 bacteria). **p<0.01, (unpaired *t* test).

Because EHEC Tir and EspF_U_ trigger pedestal assembly and increase macrocolony size, we speculated that disrupting pedestal formation on the host side by inhibiting the Arp2/3 complex could diminish colonization in a way that resembled what was seen with the EHEC effector mutants. To test this, we examined the effects of treating Caco-2 cells with the Arp2/3 complex inhibitors CK666 and CK869 during infection with KC12+EspF_U_. The inhibitors did not alter the morphology of tight junctions or microvilli on Caco-2 cells, but did interfere with pedestal assembly as expected (S5A–S5F Fig). In addition, the Arp2/3 complex inhibitors restricted the size of KC12+EspF_U_ macrocolonies compared to DMSO-treated controls (Fig 6A and 6B). To determine if this inhibition could be caused by limiting actin based motility, mCherry-actin expressing NIH3T3 cells were treated with DMSO or CK666/CK869, and motility was measured. As expected, KC12+EspF_U_ moved at less than half the speed on CK666/CK869 treated cells as on DMSO treated cells (Fig 6C). Taken together with the smaller macrocolony size and slower motility exhibited by EspF_U_-deficient bacteria, these data are consistent with the idea that actin-based motility driven by EHEC Tir and EspF_U_ plays a key role in the extent of tissue colonization.

### KC12+EspF_U_ moves faster than EPEC Y474* on polarized cells and uses motility to spread from cell-to-cell

**Fig 7:**
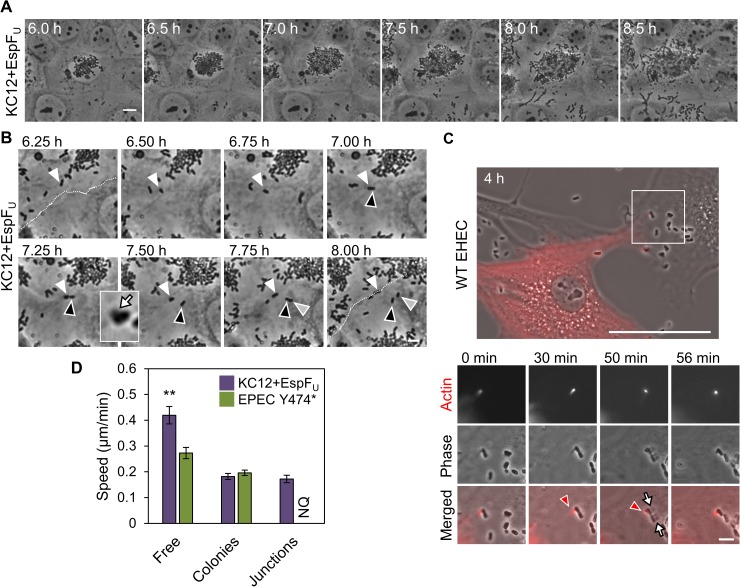
KC12+EspF_U_ moves faster than EPEC Y474* on polarized cells and uses motility to spread efficiently from cell-to-cell. (A) JEG-3 cells were infected with KC12+EspF_U_ for 6 h and imaged for 2.5 h using phase-contrast microscopy. Selected frames (taken from S2 Video) show the expansion of a macrocolony. Scale bar, 10 μm. (B) Additional images (from S2 Video) show a cell-cell junction (white dashed line), and a bacterium (white arrowhead) traveling on a pedestal (white arrow in inset). The highlighted bacterium divided, with one daughter adhered to the adjacent cell (black arrowhead). The bacterium then divided again on the same cell (gray arrowhead). Scale bar, 5 μm. (C) NIH3T3 cells expressing mCherry-actin were infected with WT EHEC for 4 h and imaged live. Selected frames from live imaging show a bacterium on an mCherry-actin pedestal (red arrowhead) reaching a nearby cell and dividing. Phase-dense pedestals can be seen on the second cell for both bacteria (white arrows), while only one possesses an mCherry-actin pedestal still connected to the original cell. Scale bar, 50 μm, inset 5 μm. (D) Bacteria were categorized as free, in macrocolonies, or at junctions based on their localization (NQ = not quantifiable). Each bar represents the mean speed (+/- SE) for 30 bacteria for each condition, with the exception of junction bacteria, where n = 15 bacteria. ** p<0.01 (ANOVA, Tukey post-hoc tests).

Although our data implicate Tir and EspF_U_ in spreading infection across a cell monolayer, this process has never been directly visualized. To better understand the mechanism of colony growth, we performed live cell imaging to monitor macrocolony development over time. JEG-3 cells were utilized as host cells because they polarize more rapidly than Caco-2 cells and are easier to image by phase-contrast microscopy. Tracking macrocolony growth over time revealed that colony expansion is partially driven by bacterial replication and spreading outwards towards cell junctions, thereby promoting infection of neighboring cells (Fig 7A, S2 Video). Further time lapse analyses (S2 Video) revealed that KC12+EspF_U_ bacteria often migrated out of colonies and eventually reached cell junctions. Bacteria moving on pedestals appeared to pause at these junctions, where they replicated to cause infection of the neighboring cell (Fig 7B). To visualize this process in more detail, and verify that EHEC employs the same mechanism of transmission, NIH3T3 fibroblasts expressing mCherry-actin were infected with WT EHEC and imaged live (Fig 7C). A bacterium moving on a pedestal containing mCherry-actin (Fig 7C, red arrowhead) contacted a nearby cell that was not expressing mCherry-actin. This difference in mCherry-actin expression allowed us to distinguish the pedestals between the two host cells, as phase-contrast microscopy revealed that the bacterium with an mCherry-labeled pedestal formed a phase-dense pedestal on the second cell (Fig 7C, top arrow) and divided, while maintaining the original pedestal. After division, one bacterium had two pedestals on the two different cells, while the other daughter was only associated with the second cell via a phase-dense pedestal (Fig 7C, bottom arrow). These observations provide the first evidence for how extracellular EPEC/EHEC spread beyond initially infected cells while remaining intimately attached to the plasma membrane.

In the course of these studies, we noticed that adherent bacteria exhibited different movement behaviors based on their associations with colonies or junctions, so we quantified motility of KC12+EspF_U_ within macrocolonies, at cell-cell junctions, or “free,” (not in colonies or at junctions). Free bacteria moved significantly faster than bacteria in tightly-packed colonies or at junctions (Fig 7D). In an attempt to uncover why EPEC colonies do not grow as large on polarized cells as KC12 macrocolonies, EPEC movement was also assessed. While KC12+EspF_U_ and EPEC Y474* bacteria in macrocolonies displayed similar speeds (Fig 7D), the frequency of EPEC Y474* reaching junctions and replicating between 6 and 8 h of infection was too low to be quantified (S3 Video). Surprisingly, and in contrast to fibroblast data (Fig 2), the population of free EPEC Y474* moved significantly slower than free KC12+EspF_U_ (Fig 7D). The faster speed of KC12+EspF_U_ provides one explanation for why these bacteria are better able to migrate out of bacterial clusters, move to junctions, and infect more cells than EPEC Y474*. Thus, EHEC Tir and EspF_U_ confer an advantage over EPEC Tir in motility and cell-to-cell spreading that is specific to polarized epithelia.

### EspF_U_-dependent actin pedestals allow for an efficient pathway of cell-to-cell transmission

**Fig 8:**
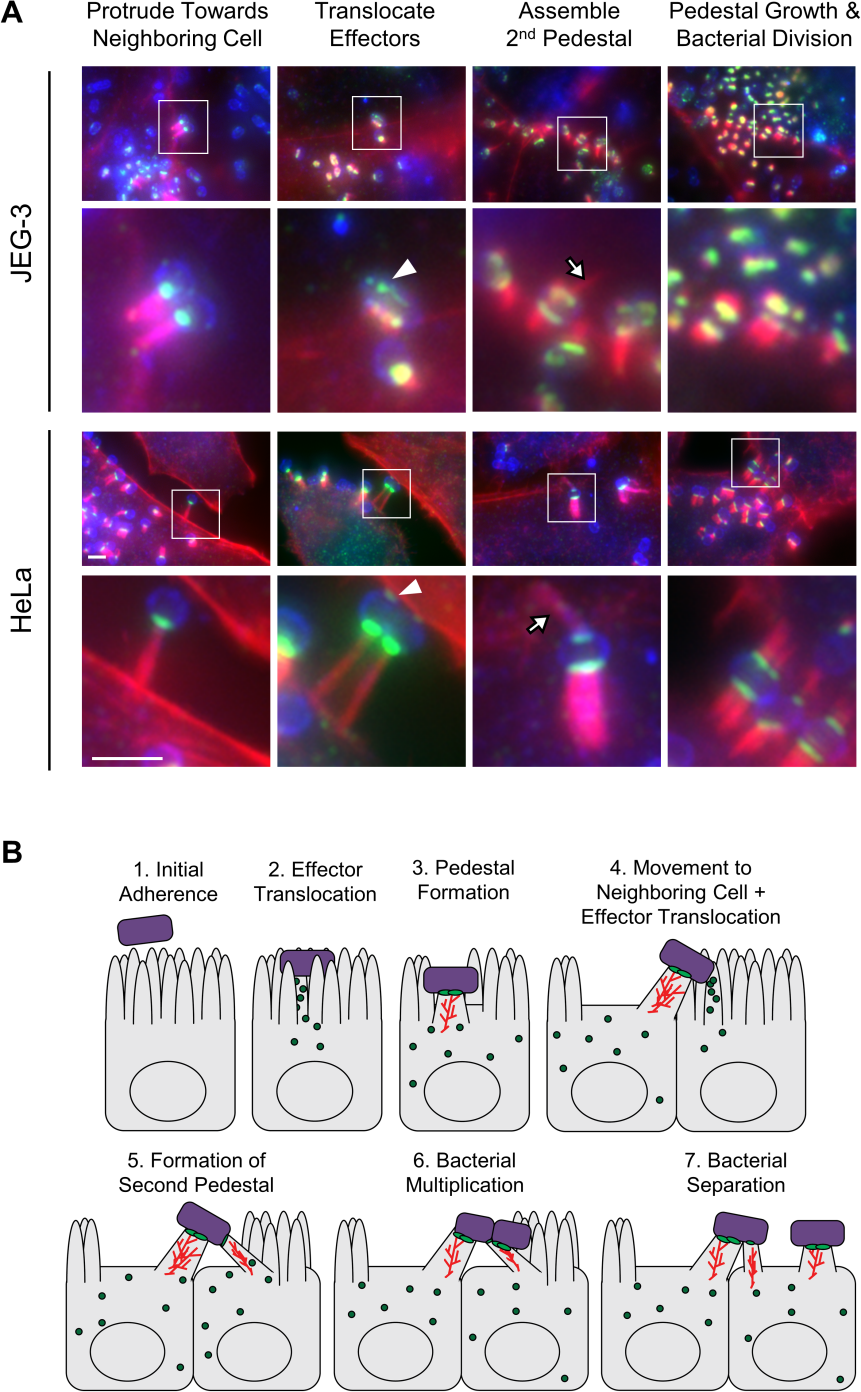
EspF_U_-dependent actin pedestals allow for an efficient pathway of cell-to-cell transmission. (A) JEG-3 and HeLa cells infected for 6 h and 5 h, respectively, were stained to show bacteria (blue), F-actin (red), and HA-Tir (green). Individual events were ordered into the following sequence based on live imaging in Fig 7: bacteria (i) use pedestals to protrude and contact an uninfected neighboring cell, (ii) translocate effectors including Tir (arrowheads) into the second cell, (iii) polymerize a second pedestal (arrows), and (iv) use the secondary pedestal to dock bacteria at junctions as the bacteria divide. Scale bars, 3 μm. (B) The proposed model is based on data from experiments in Figs 7 and 8, and is shown to incorporate every step of the infectious life cycle. Green circles represent translocated effectors, red lines indicate F-actin in pedestals, and green ovals represent Tir.

To better elucidate how cell-to-cell spreading occurs when KC12+EspF_U_ pauses at a cell junction, we fixed infected JEG-3 cells and stained for bacteria, F-actin, and HA-Tir. Additionally, we infected, fixed, and stained a highly susceptible HeLa cell line that was seeded at an optimal density for easily distinguishing the boundaries between cells. These experiments allowed us to outline a straightforward pathway for cell-to-cell transmission. We detected (1) bacteria on pedestals protruding towards neighboring cells, (2) bacteria with a pedestal on one cell and Tir in a neighboring cell, (3) bacteria with two pedestals on two different cells, and (4) bacteria that had divided and maintained pedestals on two different cells (Fig 8A). Ordering these events, and pairing them with live imaging data, we infer that the bacteria use actin-based surfing motility to escape densely-packed bacterial communities, move towards junctions with uninfected cells, contact and inject effectors into the adjacent cell, polymerize a second pedestal, divide, and eventually disengage from the first cell to complete the infection cycle by moving onto a new cell (Fig 8B).

## Discussion

EPEC and EHEC pedestals were discovered decades ago, but our understanding of their cellular functions has remained elusive. Early on, it was hypothesized that pedestals act as an anchor to maintain attachment to the host cell via direct cytoskeletal linkage [35], or that intimate adherence via Tir and intimin prevents phagocytosis [34]. More recent studies have focused on the importance of Tir signaling in intestinal colonization [40], or of actin polymerization in maintaining cell attachment and thereby enhancing type three translocation [39, 41]. It has also been speculated that surfing on pedestals could aid in the spreading of infection and the development of bacterial niches [33]. However, live examinations of EPEC and EHEC infections are limited [36, 37, 51], so how actin-based surfing motility could contribute to pathogenesis was not clear. The studies and speculations thus far have also not directly considered functional differences between the EHEC and EPEC mechanisms of pedestal assembly, so it was unknown if either of the two actin polymerization pathways have distinct advantages. In the current work, we defined similar roles for EHEC and EPEC pedestals in resisting phagocytosis and promoting motility on fibroblasts. Most importantly, we uncovered differences in colonization of polarized epithelial cells and delineated a pedestal-based mechanism of cell-to-cell transmission by EHEC.

It is well established that EPEC and EHEC are able to resist phagocytosis, and several effector proteins such as EspF, EspB, EspH, and EspJ have been implicated directly or indirectly in this capacity [34, 53–58]. EPEC strains lacking only Tir were previously shown to be phagocytosed less than a T3SS mutant, supporting a pedestal-independent mechanism of phagocytotic evasion [53]. In contrast to these results, we found that the capacity to polymerize actin into pedestals positively correlates with resistance to phagocytosis, and that pedestal deficient strains were phagocytosed at the same level as a T3SS mutant, suggesting that translocated effectors previously proposed to play an anti-phagocytic role may not be able to exert their effects in the absence of a pedestal. Our results also indicate that both the EspF_U_-mediated and phospho-Tir-mediated pathways of actin polymerization confer the same level of resistance to phagocytosis, agreeing with the common belief that similar form yields similar function for pedestals.

Pathogen motility assays on fibroblasts also revealed conserved pedestal functions, as both KC12+EspF_U_ and EPEC Y474* were capable of undergoing surfing motility at average speeds of approximately 0.35 μm/min, and maximum speeds of 4.2 μm/min. Although these are much slower than the actin-based motility rates of intracellular pathogens such as *Listeria* and *Shigella*, which average 12–36 μm/min depending on the experimental system [1, 59, 60], the surfing motility of KC12+EspF_U_ and EPEC Y474* allows for an exploration of the cell surface that surpasses that of pedestal deficient strains. Surprisingly, motility on polarized epithelial cells differed between EspF_U_-driven and phospho-Tir-driven actin polymerization, with KC12+EspF_U_ moving significantly faster than EPEC Y474*. Therefore, it is tempting to speculate that the EspF_U_ mechanism of pedestal assembly may somehow produce more force to enable better movement, specifically on polarized cells.

Studying colonization kinetics on polarized epithelial cells exposed another significantly different outcome of the EHEC and EPEC pedestal assembly pathways, as we were able to characterize the phenomenon of macrocolony formation. These extracellular infection foci are reminiscent of the plaques formed by intracellular *Listeria* and *Shigella* [61, 62]. For the *E*. *coli* strains, discrete macrocolonies grew over time and encompassed more cells. However, KC12+EspF_U_ colonies grew significantly faster and larger, covering more host cells than EPEC Y474*. Additionally, our data show that the introduction of EspF_U_ into WT EPEC can enable those bacteria to also form larger colonies. Interestingly, the size of macrocolonies is positively correlated with the speed of surfing motility on polarized cells, with a faster strain able to produce larger colonies. This is the first evidence that speed may be related to cell-to-cell spreading for pathogenic *E*. *coli*.

Intracellular pathogens that manipulate the actin cytoskeleton derive a clear advantage from actin based motility, as it promotes cell-to-cell transmission without exposure to the extracellular immune system [1, 2]. However, whether surface-associated EPEC and EHEC could benefit from surfing had never been explored, and no mechanisms for cell-to-cell spread have been proposed. How EHEC infect the intestine so efficiently has been a long-standing question, and our data provide new insight into colonization by describing how EHEC solves the complex problem of cell-to-cell transmission. Live imaging and fluorescence microscopy revealed that KC12+EspF_U_ use faster surfing motility to leave macrocolonies and reach cell junctions, where they contact a neighboring cell, translocate effectors, polymerize a second pedestal, and divide. This mechanism of stepwise cellular spread across the host intestinal tissue allows the bacteria to remain attached to the epithelial surface at all times, which would effectively reduce the risk of washing away during intestinal contractions and diarrhea.

Consistent with these results, disease models using infant rabbits and gnotobiotic piglets have demonstrated that EHEC which express EspF_U_ expand to greater numbers in the intestine than EHEC lacking EspF_U_ [42]. Intriguingly, the discrete colonization patterns of EHEC that were observed in piglet intestines *in vivo* [42] closely resemble the macrocolonies that we visualized on polarized monolayers *in vitro*. These observations, combined with the finding that KC12+EspF_U_ forms larger colonies than EPEC Y474*, raises the question of whether the advantage conferred by EspF_U_ would manifest in an *in vivo* competition assay. However, EspF_U_ from WT EHEC is able to trans-complement EspF_U_-deficient bacteria during co-infection in rabbits and on HeLa cells [42]. Similarly, we found that EspF_U_ injected by EHEC can incorporate into EPEC pedestals during co-infections of polarized monolayers *in vitro* (S4 Fig). Thus, deciphering the distinct benefits of EHEC EspF_U_ *in vivo* will require more innovative approaches in the future.

Finally, our findings prompt the question of how EHEC Tir and EspF_U_ cause faster motility and macrocolony expansion than EPEC Tir. It is plausible that EspF_U_ is a more potent activator of N-WASP than Nck1 and Nck2 because it is better at multimerizing N-WASP. Canonical EspF_U_ has six 47-residue repeats, each with the potential to recruit the N-WASP-WIP complex via a short α-helix that activates N-WASP through binding its autoinhibitory region [29–31]. Each repeat also possesses proline-rich motifs that bind the SH3 domains of IRTKS, IRSp53, and TOCA-1 to allow recruitment to Tir or enhanced N-WASP activation [25, 26, 63]. *In vitro*, each individual repeat can contribute to faster polymerization, although only two are required for efficient pedestal formation in cells [29, 31, 64]. In contrast, the Nck adaptor proteins recruited by EPEC Tir each possess three SH3 domains, all of which are able to bind the proline rich domain of N-WASP [21, 65], and include one that can bind to WIP [66]. These differences in ability to multimerize N-WASP may impact the structure or function of the pedestal, as multivalency has been shown to promote phase separation both in cells and using purified proteins [67–69]. Since increasing valency within a system results in phase separation at lower concentrations [68], EspF_U_ may promote phase transitions at lower concentrations than would be necessary for Nck1/2, leading to more efficient signaling to the Arp2/3 complex. It also remains possible that the differential recruitment of several host proteins by EHEC Tir and EspF_U_ versus EPEC Tir significantly affects motility and cell-to-cell spreading. Future work will likely shed light on how EHEC’s mechanism of pedestal formation provides motility, colonization, and transmission advantages over EPEC’s signaling pathway, and whether EPEC Tir provides its own set of benefits.

## Materials and methods

### Bacterial and mammalian cell culture

All bacterial strains are described in S1 Table [22, 27, 70–72]. In each pedestal-proficient bacterium, the main pedestal-driving effector is encoded behind the effector’s own promoter on a low copy number plasmid [22, 27]. 24 h prior to all infections, bacterial cultures were inoculated from single colonies into LB broth with appropriate antibiotics and grown shaking at 37°C for 8–9 h. Cultures were then diluted 1:500 in DMEM + 100 mM HEPES, pH 7.4, and grown standing overnight at 37°C + 5% CO_2_ to enhance the production of the T3SS and its effectors.

HeLa (University of Massachusetts Medical School) [27] and JEG-3 cells (American Type Culture Collection: ATCC) were each maintained as subconfluent monolayers in DMEM supplemented with 10% fetal bovine serum (FBS) and antibiotic/antimycotic. HeLa cells were seeded at approximately 5x10^5^ cells/ml onto glass coverslips 24 h before infections, while JEG-3 cells were seeded at approximately 5x10^5^ cells/ml either onto glass coverslips or into 35 mm glass-bottom dishes (MatTek) and allowed to grow for 24–48 h post confluency. THP-1 monocytes (ATCC) were grown in suspension in RPMI 1640 supplemented with 10% FBS, antibiotic/antimycotic, and 0.05 mM 2-mercaptoethanol. Cells seeded at a density 2x10^5^ cells/ml were activated to a macrophage-like state by adding 40 ng/ml phorbol 12-myristate 13-acetate (PMA) to the media, and allowing the cells to adhere to glass coverslips in 24-well plates for approximately 72 h [73] before infections. NIH3T3 cells (University of California Berkeley, cell culture facility) stably expressing mCherry-βactin [74] were maintained in DMEM supplemented with 10% FBS, antibiotic/antimycotic, and 500 μg/ml G418. 48 h before infection, cells were seeded at approximately 5x10^5^ cells/ml onto 35 mm glass-bottom dishes in media lacking G418, and 16 h prior to infection were induced to express mCherry-actin with 7.6 mM sodium butyrate. C2BBe1 (referred to as Caco-2) cells (ATCC) were maintained in subconfluent monolayers in DMEM supplemented with 10% FBS, antibiotic/antimycotic, and 0.01 mg/ml human transferrin. To create polarized monolayers [75], cells were grown to confluency on glass coverslips or aclar (for EM) in 24-well plates, and given half media changes every 1–2 days for at least 2 weeks prior to infections. All cells were grown at 37°C + 5% CO_2_.

### Infections and chemical inhibitors

For phagocytosis assays, activated THP-1 cells were washed twice with warm phosphate buffered saline (PBS) and infected with bacteria diluted 1:1000 in RPMI + 3.5% FBS + 20mM HEPES to achieve a multiplicity of infection (MOI) of ~3. Plates were centrifuged at 172 x *g* for 5 min to synchronize infections. Infected cells were incubated at 37°C + 5% CO_2_ for 15–150 min. At various time points, cells were washed 3 times with warm PBS and fixed for 20 min using 2.5% paraformaldehyde (PFA) prior to performing outside-inside staining, or fixed for 5 min in methanol prior to performing LAMP-1 staining.

For bacterial motility assays, NIH3T3 cells were washed twice with PBS and infected with bacteria diluted 1:200 into DMEM + 3.5% FBS + 20 mM HEPES to achieve an MOI of ~6. Infected cells were rotated for 5 min to disperse the bacteria before incubation for 3–5 h. Cells were then washed twice with PBS, given fresh media, and imaged for 2–3 h at 26°C in an environmental chamber (Okolab). Live imaging with Arp2/3 complex inhibitors [76] was performed by adding 50 μM CK666 + 50 μM CK869 (Calbiochem) or equivalent doses of DMSO. The addition of DMSO or Arp2/3 complex inhibitors did not restrict bacterial growth compared to media alone. JEG-3 cells were imaged at 37°C between 3 and 8 h post infection.

For colonization assays, polarized Caco-2 monolayers were washed twice and infected with overnight cultures diluted 1:33 in DMEM + 3.5% FBS + 20 mM HEPES for an MOI of ~10. CFU counts were performed to verify equal input for all bacterial strains. Plates were centrifuged and subjected to PBS washes and media changes every 1 h to remove unbound bacteria. After 6 h, cells were washed 3 times with PBS, and fixed with 3.7% PFA for 30 min. Colonization assays using EHEC strains were infected as described above, but PBS washes and media changes were performed every 2 h, and the total infection was carried out for 8 h. Experiments involving Arp2/3 inhibitors were completed by pretreating monolayers for 15 min with 50 μM CK666 + 50 μM CK869 and using fresh media with inhibitors throughout the infection process.

### Fluorescence microscopy

For standard immunofluorescence, fixed cells were washed 3 times with PBS, permeabilized for 2 min with 0.1% TritonX-100, washed 3 times with PBS, and blocked using 1% FBS + 1% bovine serum albumin (BSA) in PBS + 0.02% NaN_3_ for a minimum of 30 min. Cells were probed with primary antibodies diluted in blocking buffer at concentrations listed in S2 Table for 35–40 min. Cells were then washed 3 times with PBS and treated with secondary antibodies and/or DAPI and phalloidin for 35 min, at concentrations listed in S2 Table. Cells were then washed 3 times with PBS, and mounted in Prolong Gold anti-fade. For outside-inside staining, fixed THP-1 cells were blocked in 1% BSA + 1% FBS + 1% normal goat serum (NGS) + 0.02% NaN_3_ in PBS for 30 min. Cells were incubated in mouse anti-LPS for 40 min, followed by 3 PBS washes, and Alexa 555 anti-mouse antibodies for 40 min, followed by 3 PBS washes. Cells were then permeabilized with 0.1% Triton X-100 for 2 min, washed 3 times, and re-blocked for 30 min. The same primary antibody was used again for 40 min, but Alexa488 anti-mouse was used as a secondary antibody for 40 min, and cells were also stained with DAPI, similar to previous experiments [53]. After washing, coverslips were mounted in Prolong Gold anti-fade. All fixed and live cells were imaged using a Nikon Eclipse T*i* microscope equipped with a Plan Apoλ 100x 1.45 NA objective, a Plan Fluor 20x 0.5 NA objective, an Andor Clara-E camera, and a computer running NIS Elements software. Image analyses were performed using ImageJ. Live phase-contrast imaging as well as mCherry and GFP fluorescence of infected NIH3T3 cells was performed using the 100x objective, and images were captured at 30 s intervals. Live imaging of JEG-3 cells was performed using a 20x phase-contrast objective, and images were acquired at 30 or 45 s intervals.

### Electron microscopy

Caco-2 cells were fixed in 2% glutaraldehyde + 2.5% paraformaldehyde in 0.1 M Na cacodylate buffer [51] + 1.5 mM CaCl_2_ + 1.5 mM MgCl_2_, pH 7.4 with 0.2% tannic acid [77]. The primary fixation was carried out for 15 min at room temperature, followed by replacement with fresh fixative and incubation for 1 h at room temperature. Monolayers were washed in 0.1 M Na cacodylate buffer + 1.5 mM CaCl_2_ + 1.5 mM MgCl_2_, pH 7.4 twice for 20 min, and a third time overnight at 4°. Osmium postfixation was performed on all samples using 1% Osmium tetroxide [51] and 0.8% K_3_Fe(CN)_6_ in 0.1 M Na cacodylate buffer + 1.5 mM CaCl_2_ + 1.5 mM MgCl_2_, pH 7.4. Samples were rinsed twice with distilled water for 15 min, and transmission electron microscopy (TEM) samples were subjected to *en bloc* fixation in 1% aqueous uranyl acetate [51] for 80 min, followed by two 10 min washes. All samples were dehydrated through a graded ethanol series (30%, 50%, 70%, 95%, 100%, 100%) using 10 min wash times, and scanning electron microscopy (SEM) samples were then pulled for critical point drying. TEM samples continued on to 2 10 min acetone washes and were embedded in Embed 812 + Araldite 506 + DDSA + 1.5% DMP-30 by using 1:1 resin:acetone for 90 min, 3:1 resin:acetone for ~15 h and 100% resin twice in 2 h incubation steps. Resin was polymerized in a 60°C oven for 49 h. Aclar was removed from the resin, and the tissue was reembedded in resin for 49 h. 100 nm ultrathin sections were cut using a diamond knife on a Leica microtome. Sections were lifted onto 200-mesh copper grids, and stained for 8 min with 2% aqueous uranyl acetate, followed by 2 min in 2.5% lead citrate. TEM was carried out using a Technai Spirit electon microscope, operated at 80 kV. SEM was performed using a FEI Nova NanoSEM 450 microscope.

### Data analysis and statistics

Motility assays were quantified using ImageJ software. Movement was tracked with the MTrackJ plugin, and tracking plots were generated using the chemotaxis tool plugin. Colony sizes were measured using the threshold feature followed by the analyze particles feature. In most cases, the lower limit of colony sizes was set to 100 μm^2^. Exceptions included time courses with short time points, the use of inhibitors, or experiments which included strains that were severely deficient at colony formation (KC12Δ*tir* strains), which required lowering the limit to 50 or 25 μm^2^. Statistics on data sets with 3 or more conditions were performed using one-way ANOVAs followed by Tukey’s post-hoc test unless otherwise indicated. P-values for data sets including 2 conditions were determined using unpaired *t* tests unless otherwise noted. For phagocytosis assays (S1D Fig), SE was calculated based on the number of coverslips examined. For motility quantifications in which all pedestals on a single cell were averaged (Fig 2C and 2F), SE was calculated based on the number of cells examined. For motility assays in which the mean of all pedestals was calculated (Figs 6C and 7D), SE was calculated based on the number of pedestals examined. SE values for the fraction of cells moving (Fig 2D) and for the directional persistence (Fig 2E) were calculated from the total number of pedestals, and the total number of moving pedestals, respectively. The SEs for colony size in Figs 3F and 5B were calculated based on the number of coverslips, while SDs and SEs in Fig 4B–4D were based on the number of experiments. For smaller sample sizes (Fig 4F and Fig 6B), and for Fig 5E and 5G, the number of colonies was used to generate SD and SE. Finally, quantifications of the % area covered (Fig 4E and Fig 5C) were averaged by field of view (FOV), and the total number of FOVs was used for SD and SE determination.

## Supporting information

S1 Fig The EPEC and EHEC mechanisms of pedestal assembly both provide anti-phagocytic functions. (A) Activated THP-1 macrophages were infected for 3.5 h, fixed, and stained to visualize DNA, HA-Tir, and F-actin. Scale bar, 10 μm. (B) Activated THP-1 cells infected for 90 min were differentially stained to determine the total number of cell-associated bacteria (red), and the number of external bacteria (green). Scale bar, 10 μm. (C) Activated THP-1 cells infected for 90 min with EPEC were fixed and stained for DNA, bacteria, and LAMP-1. Scale bar, 10 μm; inset scale bar, 1 μm. (D) The % of internalized bacteria was quantified at the depicted times. Each data point represents the mean (+/- SE) calculated from 4–6 coverslips with 200–300 total cells spanning at least 3 experiments. **p<0.01, *** p<0.001 (ANOVA, Tukey post-hoc tests).
(TIF)

S2 Fig KC12+EspF_U_ disrupts microvilli of polarized cells, but does not significantly alter tight junctions. (A) HeLa, JEG-3, or Caco-2 cells were left uninfected or infected with KC12+EspF_U_ for 6 h. Cells were fixed and stained with phalloidin to detect F-actin, Ezrin antibodies to stain microvilli, ZO-1 antibodies to visualize tight junctions, and DAPI to label DNA. Scale bar, 25 μm. (B) Polarized Caco-2 monolayers were infected with KC12+EspF_U_ for 6 h, fixed, and stained to detect Ezrin (top) or ZO-1 (bottom), in addition to bacterial LPS, DNA, and F-actin. Areas of low (first and third rows) and high (second and fourth rows) bacterial burdens were imaged from the same coverslip for each staining condition. Scale bar, 50 μm.
(TIF)

S3 Fig KC12 and EPEC strains divide at similar rates in suspension and on cells. (A) Bacteria grown in infection media were diluted and plated every 90 min to determine the number of Colony Forming Units (CFUs). Each data point represents the mean number of CFUs (+SD) from 4 experiments. (B) JEG-3 cells were infected for 6 h with the indicated strains and imaged live. Individual bacteria were tracked over time to determine the amount of time between consecutive divisions and calculate the maximum division rate. Each point represents a single bacterium, with the mean (+/- SD) indicated in black.
(TIF)

S4 Fig EspF_U_ and Tir can colocalize even if delivered by independent bacteria. (A) Polarized Caco-2 monolayers were infected with EPEC+EspF_U_ or KC12+EspF_U_, fixed and stained for EspF_U_-myc, F-actin, and DNA. Scale bar, 10 μm. (B) JEG-3 monolayers were co-infected for 6 h with equal amounts of EPEC Y474* and EHECΔ*espF*_*U*_+EspF_U_, fixed, and stained for HA-Tir (which is only tagged in EPEC), EspF_U_-myc (which is only expressed by EHEC), F-actin, and DNA. Areas of isolated EHEC bacteria (i), isolated EPEC bacteria (ii), or mixed infection (iii) are shown in insets. Colocalization between HA-Tir and EspF_U_-myc is highlighted with arrowheads, indicating that bacteria can share pedestal effectors during co-infection. Scale bars, 50 μm, inset 5 μm. (C) JEG-3 monolayers were co-infected and fixed as in (B), but stained for EHEC O157 in addition to HA-Tir. Colocalization between HA-Tir and O157 was not observed, suggesting that Tir is not effectively transferred from EPEC to EHEC. Scale bars, 50 μm, inset 12.5 μm. (D) JEG-3 monolayers were co-infected with EPEC Y474* and EPEC+EspF_U_, fixed, and stained for HA-Tir (which is only tagged in EPEC Y474*) and EspF_U_-myc (which is only expressed in EPEC+EspF_U_). Colocalization indicates that EPEC strains can efficiently share HA-Tir and EspF_U_. Scale bar, 5 μm.
(TIF)

S5 Fig Arp2/3 inhibitors do not disrupt microvilli or tight junctions, but do inhibit actin pedestal formation. (A-B) Polarized Caco-2 monolayers were treated with either DMSO or CK666+CK869 for 6 h, fixed, and stained for DNA, F-actin, and either Ezrin or ZO-1. Scale bar, 50 μm. (C) Polarized Caco-2 monolayers were pretreated for 15 min with DMSO or CK666+CK869, then infected for 6 h with KC12+EspF_U_ in the presence of either DMSO or inhibitors. Cells were fixed and stained for F-actin and bacteria. Scale bar, 10 μm. (D) NIH3T3 cells were infected with KC12+EspF_U_ and treated with DMSO or CK666+CK869 for 4 h, fixed, and stained for HA-Tir, F-actin, and bacteria. Scale bar, 10 μm. (E) The % of HA-Tir positive foci that were associated with actin pedestals from experiments in (D) was calculated. Each bar represents the mean (+/- SE) calculated from 15 cells, each harboring up to 50 bacteria. (F) The relative intensity of pedestals associated with HA-Tir foci was calculated and normalized to an adjacent pedestal-free area of the cell that was set to 1. Each bar represents the mean (+/- SE) calculated from 9 cells harboring up to 40 pedestals per cell. *p<0.05, ***p<0.001 (unpaired *t* tests).
(TIF)

S1 Table Strains used in this study. (PDF)

S2 Table Antibodies and molecular probes used in this study. (PDF)

S1 Video EPEC exhibits surfing motility. NIH3T3 cells stably expressing mCherry-actin (red) were infected with EPEC+GFP (green) for 3 h prior to imaging. Images were acquired every 30 s using a 100x objective, and processed in ImageJ. Playback is at 10 frames/s. Scale bar, 10 μm.
(AVI)

S2 Video Actin pedestals formed by KC12+EspF_U_ promote infection of neighboring cells. JEG-3 monolayers were infected with KC12+EspF_U_ for 6 h prior to imaging at 37°C. Images were acquired every 45 s using a 20x phase-contrast objective, and processed in imageJ. Playback is at 20 frames/s. Inset movies show (i) a macrocolony expanding, (ii) KC12+EspF_U_ bacteria paused at a junction, and (iii) bacteria replicating at a junction and infecting the neighboring cell.
(AVI)

S3 Video Actin pedestals formed by EPEC Y474* move slowly on JEG-3 cells. JEG-3 monolayers were infected with EPEC Y474* for 6 h prior to imaging at 37°C. Images were acquired every 30 s using a 20x phase-contrast objective and processed in imageJ. Playback is at 30 frames/s. The inset movie shows a macrocolony.
(AVI)

## References

[1] Welch MD, Way M. Arp2/3-mediated actin-based motility: a tail of pathogen abuse. Cell Host Microbe. 2013;14(3):242–55. doi: 10.1016/j.chom.2013.08.011 ; PubMed Central PMCID: PMCPMC3933244. 24034611 10.1016/j.chom.2013.08.011 PMC3933244

[2] Kuehl CJ, Dragoi AM, Talman A, Agaisse H. Bacterial spread from cell to cell: beyond actin-based motility. Trends Microbiol. 2015;23(9):558–66. doi: 10.1016/j.tim.2015.04.010 ; PubMed Central PMCID: PMCPMC4560970. 26021574 10.1016/j.tim.2015.04.010 PMC4560970

[3] Ireton K. Molecular mechanisms of cell-cell spread of intracellular bacterial pathogens. Open Biol. 2013;3(7):130079 doi: 10.1098/rsob.130079 ; PubMed Central PMCID: PMCPMC3728924. 23864553 10.1098/rsob.130079 PMC3728924

[4] Hayward RD, Leong JM, Koronakis V, Campellone KG. Exploiting pathogenic Escherichia coli to model transmembrane receptor signalling. Nat Rev Microbiol. 2006;4(5):358–70. doi: 10.1038/nrmicro1391 . 16582930 10.1038/nrmicro1391

[5] Moon HW, Whipp SC, Argenzio RA, Levine MM, Giannella RA. Attaching and effacing activities of rabbit and human enteropathogenic Escherichia coli in pig and rabbit intestines. Infect Immun. 1983;41(3):1340–51. ; PubMed Central PMCID: PMCPMC264644. 6350186 10.1128/iai.41.3.1340-1351.1983 PMC264644

[6] Knutton S, Baldwin T, Williams PH, McNeish AS. Actin accumulation at sites of bacterial adhesion to tissue culture cells: basis of a new diagnostic test for enteropathogenic and enterohemorrhagic Escherichia coli. Infect Immun. 1989;57(4):1290–8. ; PubMed Central PMCID: PMCPMC313264. 2647635 10.1128/iai.57.4.1290-1298.1989 PMC313264

[7] Donnenberg MS, Tzipori S, McKee ML, O'Brien AD, Alroy J, Kaper JB. The role of the eae gene of enterohemorrhagic Escherichia coli in intimate attachment in vitro and in a porcine model. J Clin Invest. 1993;92(3):1418–24. doi: 10.1172/JCI116718 ; PubMed Central PMCID: PMCPMC288286. 8376595 10.1172/JCI116718 PMC288286

[8] Tzipori S, Gunzer F, Donnenberg MS, de Montigny L, Kaper JB, Donohue-Rolfe A. The role of the eaeA gene in diarrhea and neurological complications in a gnotobiotic piglet model of enterohemorrhagic Escherichia coli infection. Infect Immun. 1995;63(9):3621–7. ; PubMed Central PMCID: PMCPMC173502. 7642299 10.1128/iai.63.9.3621-3627.1995 PMC173502

[9] Marches O, Nougayrede JP, Boullier S, Mainil J, Charlier G, Raymond I, et al Role of tir and intimin in the virulence of rabbit enteropathogenic Escherichia coli serotype O103:H2. Infect Immun. 2000;68(4):2171–82. ; PubMed Central PMCID: PMCPMC97401. 10722617 10.1128/iai.68.4.2171-2182.2000 PMC97401

[10] Deng W, Vallance BA, Li Y, Puente JL, Finlay BB. Citrobacter rodentium translocated intimin receptor (Tir) is an essential virulence factor needed for actin condensation, intestinal colonization and colonic hyperplasia in mice. Mol Microbiol. 2003;48(1):95–115. . 12657048 10.1046/j.1365-2958.2003.03429.x

[11] Ritchie JM, Thorpe CM, Rogers AB, Waldor MK. Critical roles for stx2, eae, and tir in enterohemorrhagic Escherichia coli-induced diarrhea and intestinal inflammation in infant rabbits. Infect Immun. 2003;71(12):7129–39. ; PubMed Central PMCID: PMCPMC308950. doi: 10.1128/IAI.71.12.7129-7139.2003 14638803 10.1128/IAI.71.12.7129-7139.2003 PMC308950

[12] Schuller S, Chong Y, Lewin J, Kenny B, Frankel G, Phillips AD. Tir phosphorylation and Nck/N-WASP recruitment by enteropathogenic and enterohaemorrhagic Escherichia coli during ex vivo colonization of human intestinal mucosa is different to cell culture models. Cell Microbiol. 2007;9(5):1352–64. doi: 10.1111/j.1462-5822.2006.00879.x . 17474908 10.1111/j.1462-5822.2006.00879.x

[13] Donnenberg MS, Tacket CO, James SP, Losonsky G, Nataro JP, Wasserman SS, et al Role of the eaeA gene in experimental enteropathogenic Escherichia coli infection. J Clin Invest. 1993;92(3):1412–7. doi: 10.1172/JCI116717 ; PubMed Central PMCID: PMCPMC288285. 8376594 10.1172/JCI116717 PMC288285

[14] Tacket CO, Sztein MB, Losonsky G, Abe A, Finlay BB, McNamara BP, et al Role of EspB in experimental human enteropathogenic Escherichia coli infection. Infect Immun. 2000;68(6):3689–95. ; PubMed Central PMCID: PMCPMC97660. 10816529 10.1128/iai.68.6.3689-3695.2000 PMC97660

[15] Wong AR, Pearson JS, Bright MD, Munera D, Robinson KS, Lee SF, et al Enteropathogenic and enterohaemorrhagic Escherichia coli: even more subversive elements. Mol Microbiol. 2011;80(6):1420–38. doi: 10.1111/j.1365-2958.2011.07661.x . 21488979 10.1111/j.1365-2958.2011.07661.x

[16] Kenny B, DeVinney R, Stein M, Reinscheid DJ, Frey EA, Finlay BB. Enteropathogenic E. coli (EPEC) transfers its receptor for intimate adherence into mammalian cells. Cell. 1997;91(4):511–20. . 9390560 10.1016/s0092-8674(00)80437-7

[17] DeVinney R, Stein M, Reinscheid D, Abe A, Ruschkowski S, Finlay BB. Enterohemorrhagic Escherichia coli O157:H7 produces Tir, which is translocated to the host cell membrane but is not tyrosine phosphorylated. Infect Immun. 1999;67(5):2389–98. ; PubMed Central PMCID: PMCPMC115983. 10225900 10.1128/iai.67.5.2389-2398.1999 PMC115983

[18] Kenny B. Phosphorylation of tyrosine 474 of the enteropathogenic Escherichia coli (EPEC) Tir receptor molecule is essential for actin nucleating activity and is preceded by additional host modifications. Mol Microbiol. 1999;31(4):1229–41. . 10096089 10.1046/j.1365-2958.1999.01265.x

[19] Phillips N, Hayward RD, Koronakis V. Phosphorylation of the enteropathogenic E. coli receptor by the Src-family kinase c-Fyn triggers actin pedestal formation. Nat Cell Biol. 2004;6(7):618–25. doi: 10.1038/ncb1148 . 15220932 10.1038/ncb1148

[20] Swimm A, Bommarius B, Li Y, Cheng D, Reeves P, Sherman M, et al Enteropathogenic Escherichia coli use redundant tyrosine kinases to form actin pedestals. Mol Biol Cell. 2004;15(8):3520–9. doi: 10.1091/mbc.E04-02-0093 ; PubMed Central PMCID: PMCPMC491815. 15155808 10.1091/mbc.E04-02-0093 PMC491815

[21] Gruenheid S, DeVinney R, Bladt F, Goosney D, Gelkop S, Gish GD, et al Enteropathogenic E. coli Tir binds Nck to initiate actin pedestal formation in host cells. Nat Cell Biol. 2001;3(9):856–9. doi: 10.1038/ncb0901-856 . 11533668 10.1038/ncb0901-856

[22] Campellone KG, Giese A, Tipper DJ, Leong JM. A tyrosine-phosphorylated 12-amino-acid sequence of enteropathogenic Escherichia coli Tir binds the host adaptor protein Nck and is required for Nck localization to actin pedestals. Mol Microbiol. 2002;43(5):1227–41. . 11918809 10.1046/j.1365-2958.2002.02817.x

[23] Kalman D, Weiner OD, Goosney DL, Sedat JW, Finlay BB, Abo A, et al Enteropathogenic E. coli acts through WASP and Arp2/3 complex to form actin pedestals. Nat Cell Biol. 1999;1(6):389–91. doi: 10.1038/14087 ; PubMed Central PMCID: PMCPMC2828053. 10559969 10.1038/14087 PMC2828053

[24] Lommel S, Benesch S, Rottner K, Franz T, Wehland J, Kuhn R. Actin pedestal formation by enteropathogenic Escherichia coli and intracellular motility of Shigella flexneri are abolished in N-WASP-defective cells. EMBO Rep. 2001;2(9):850–7. doi: 10.1093/embo-reports/kve197 ; PubMed Central PMCID: PMCPMC1084051. 11559594 10.1093/embo-reports/kve197 PMC1084051

[25] Vingadassalom D, Kazlauskas A, Skehan B, Cheng HC, Magoun L, Robbins D, et al Insulin receptor tyrosine kinase substrate links the E. coli O157:H7 actin assembly effectors Tir and EspF(U) during pedestal formation. Proc Natl Acad Sci U S A. 2009;106(16):6754–9. doi: 10.1073/pnas.0809131106 ; PubMed Central PMCID: PMCPMC2672544. 19366662 10.1073/pnas.0809131106 PMC2672544

[26] Weiss SM, Ladwein M, Schmidt D, Ehinger J, Lommel S, Stading K, et al IRSp53 links the enterohemorrhagic E. coli effectors Tir and EspFU for actin pedestal formation. Cell Host Microbe. 2009;5(3):244–58. doi: 10.1016/j.chom.2009.02.003 . 19286134 10.1016/j.chom.2009.02.003

[27] Campellone KG, Robbins D, Leong JM. EspFU is a translocated EHEC effector that interacts with Tir and N-WASP and promotes Nck-independent actin assembly. Dev Cell. 2004;7(2):217–28. doi: 10.1016/j.devcel.2004.07.004 . 15296718 10.1016/j.devcel.2004.07.004

[28] Garmendia J, Phillips AD, Carlier MF, Chong Y, Schuller S, Marches O, et al TccP is an enterohaemorrhagic Escherichia coli O157:H7 type III effector protein that couples Tir to the actin-cytoskeleton. Cell Microbiol. 2004;6(12):1167–83. doi: 10.1111/j.1462-5822.2004.00459.x . 15527496 10.1111/j.1462-5822.2004.00459.x

[29] Cheng HC, Skehan BM, Campellone KG, Leong JM, Rosen MK. Structural mechanism of WASP activation by the enterohaemorrhagic E. coli effector EspF(U). Nature. 2008;454(7207):1009–13. doi: 10.1038/nature07160 ; PubMed Central PMCID: PMCPMC2719906. 18650809 10.1038/nature07160 PMC2719906

[30] Sallee NA, Rivera GM, Dueber JE, Vasilescu D, Mullins RD, Mayer BJ, et al The pathogen protein EspF(U) hijacks actin polymerization using mimicry and multivalency. Nature. 2008;454(7207):1005–8. doi: 10.1038/nature07170 ; PubMed Central PMCID: PMCPMC2749708. 18650806 10.1038/nature07170 PMC2749708

[31] Campellone KG, Cheng HC, Robbins D, Siripala AD, McGhie EJ, Hayward RD, et al Repetitive N-WASP-binding elements of the enterohemorrhagic Escherichia coli effector EspF(U) synergistically activate actin assembly. PLoS Pathog. 2008;4(10):e1000191 doi: 10.1371/journal.ppat.1000191 ; PubMed Central PMCID: PMCPMC2567903. 18974829 10.1371/journal.ppat.1000191 PMC2567903

[32] Goosney DL, DeVinney R, Finlay BB. Recruitment of cytoskeletal and signaling proteins to enteropathogenic and enterohemorrhagic Escherichia coli pedestals. Infect Immun. 2001;69(5):3315–22. doi: 10.1128/IAI.69.5.3315-3322.2001 ; PubMed Central PMCID: PMCPMC98290. 11292754 10.1128/IAI.69.5.3315-3322.2001 PMC98290

[33] Lai Y, Rosenshine I, Leong JM, Frankel G. Intimate host attachment: enteropathogenic and enterohaemorrhagic Escherichia coli. Cell Microbiol. 2013;15(11):1796–808. doi: 10.1111/cmi.12179 ; PubMed Central PMCID: PMCPMC4036124. 23927593 10.1111/cmi.12179 PMC4036124

[34] Goosney DL, Celli J, Kenny B, Finlay BB. Enteropathogenic Escherichia coli inhibits phagocytosis. Infect Immun. 1999;67(2):490–5. ; PubMed Central PMCID: PMCPMC96346. 9916050 10.1128/iai.67.2.490-495.1999 PMC96346

[35] Goosney DL, DeVinney R, Pfuetzner RA, Frey EA, Strynadka NC, Finlay BB. Enteropathogenic E. coli translocated intimin receptor, Tir, interacts directly with alpha-actinin. Curr Biol. 2000;10(12):735–8. . 10873808 10.1016/s0960-9822(00)00543-1

[36] Sanger JM, Chang R, Ashton F, Kaper JB, Sanger JW. Novel form of actin-based motility transports bacteria on the surfaces of infected cells. Cell Motil Cytoskeleton. 1996;34(4):279–87. doi: 10.1002/(SICI)1097-0169(1996)34:4<279::AID-CM3>3.0.CO;2-3 . 8871815 10.1002/(SICI)1097-0169(1996)34:4<279::AID-CM3>3.0.CO;2-3

[37] Shaner NC, Sanger JW, Sanger JM. Actin and alpha-actinin dynamics in the adhesion and motility of EPEC and EHEC on host cells. Cell Motil Cytoskeleton. 2005;60(2):104–20. doi: 10.1002/cm.20047 . 15627283 10.1002/cm.20047

[38] Vingadassalom D, Campellone KG, Brady MJ, Skehan B, Battle SE, Robbins D, et al Enterohemorrhagic E. coli requires N-WASP for efficient type III translocation but not for EspFU-mediated actin pedestal formation. PLoS Pathog. 2010;6(8):e1001056 doi: 10.1371/journal.ppat.1001056 ; PubMed Central PMCID: PMCPMC2924363. 20808845 10.1371/journal.ppat.1001056 PMC2924363

[39] Battle SE, Brady MJ, Vanaja SK, Leong JM, Hecht GA. Actin pedestal formation by enterohemorrhagic Escherichia coli enhances bacterial host cell attachment and concomitant type III translocation. Infect Immun. 2014;82(9):3713–22. doi: 10.1128/IAI.01523-13 ; PubMed Central PMCID: PMCPMC4187837. 24958711 10.1128/IAI.01523-13 PMC4187837

[40] Crepin VF, Girard F, Schuller S, Phillips AD, Mousnier A, Frankel G. Dissecting the role of the Tir:Nck and Tir:IRTKS/IRSp53 signalling pathways in vivo. Mol Microbiol. 2010;75(2):308–23. doi: 10.1111/j.1365-2958.2009.06938.x ; PubMed Central PMCID: PMCPMC2814079. 19889090 10.1111/j.1365-2958.2009.06938.x PMC2814079

[41] Mallick EM, Garber JJ, Vanguri VK, Balasubramanian S, Blood T, Clark S, et al The ability of an attaching and effacing pathogen to trigger localized actin assembly contributes to virulence by promoting mucosal attachment. Cell Microbiol. 2014;16(9):1405–24. doi: 10.1111/cmi.12302 ; PubMed Central PMCID: PMCPMC4146666. 24780054 10.1111/cmi.12302 PMC4146666

[42] Ritchie JM, Brady MJ, Riley KN, Ho TD, Campellone KG, Herman IM, et al EspFU, a type III-translocated effector of actin assembly, fosters epithelial association and late-stage intestinal colonization by E. coli O157:H7. Cell Microbiol. 2008;10(4):836–47. doi: 10.1111/j.1462-5822.2007.01087.x ; PubMed Central PMCID: PMCPMC2504705. 18067584 10.1111/j.1462-5822.2007.01087.x PMC2504705

[43] Cantey JR, Moseley SL. HeLa cell adherence, actin aggregation, and invasion by nonenteropathogenic Escherichia coli possessing the eae gene. Infect Immun. 1991;59(11):3924–9. ; PubMed Central PMCID: PMCPMC258978. 1682254 10.1128/iai.59.11.3924-3929.1991 PMC258978

[44] Brady MJ, Radhakrishnan P, Liu H, Magoun L, Murphy KC, Mukherjee J, et al Enhanced Actin Pedestal Formation by Enterohemorrhagic Escherichia coli O157:H7 Adapted to the Mammalian Host. Front Microbiol. 2011;2:226 doi: 10.3389/fmicb.2011.00226 ; PubMed Central PMCID: PMCPMC3219212. 22102844 10.3389/fmicb.2011.00226 PMC3219212

[45] Campellone KG, Rankin S, Pawson T, Kirschner MW, Tipper DJ, Leong JM. Clustering of Nck by a 12-residue Tir phosphopeptide is sufficient to trigger localized actin assembly. J Cell Biol. 2004;164(3):407–16. doi: 10.1083/jcb.200306032 ; PubMed Central PMCID: PMCPMC2172230. 14757753 10.1083/jcb.200306032 PMC2172230

[46] Campellone KG, Leong JM. Nck-independent actin assembly is mediated by two phosphorylated tyrosines within enteropathogenic Escherichia coli Tir. Mol Microbiol. 2005;56(2):416–32. doi: 10.1111/j.1365-2958.2005.04558.x . 15813734 10.1111/j.1365-2958.2005.04558.x

[47] Gross M, Salame TM, Jung S. Guardians of the Gut—Murine Intestinal Macrophages and Dendritic Cells. Front Immunol. 2015;6:254 doi: 10.3389/fimmu.2015.00254 ; PubMed Central PMCID: PMCPMC4451680. 26082775 10.3389/fimmu.2015.00254 PMC4451680

[48] Rougerie P, Miskolci V, Cox D. Generation of membrane structures during phagocytosis and chemotaxis of macrophages: role and regulation of the actin cytoskeleton. Immunol Rev. 2013;256(1):222–39. doi: 10.1111/imr.12118 ; PubMed Central PMCID: PMCPMC3806206. 24117824 10.1111/imr.12118 PMC3806206

[49] Scaletsky IC, Silva ML, Trabulsi LR. Distinctive patterns of adherence of enteropathogenic Escherichia coli to HeLa cells. Infect Immun. 1984;45(2):534–6. ; PubMed Central PMCID: PMCPMC263286. 6146569 10.1128/iai.45.2.534-536.1984 PMC263286

[50] Mills E, Baruch K, Aviv G, Nitzan M, Rosenshine I. Dynamics of the type III secretion system activity of enteropathogenic Escherichia coli. MBio. 2013;4(4). doi: 10.1128/mBio.00303-13 ; PubMed Central PMCID: PMCPMC3735188. 23900171 10.1128/mBio.00303-13 PMC3735188

[51] Shifrin DA Jr., Crawley SW, Grega-Larson NE, Tyska MJ. Dynamics of brush border remodeling induced by enteropathogenic E. coli. Gut Microbes. 2014;5(4):504–16. doi: 10.4161/gmic.32084 . 25076126 10.4161/gmic.32084 PMC5642117

[52] Brady MJ, Campellone KG, Ghildiyal M, Leong JM. Enterohaemorrhagic and enteropathogenic Escherichia coli Tir proteins trigger a common Nck-independent actin assembly pathway. Cell Microbiol. 2007;9(9):2242–53. doi: 10.1111/j.1462-5822.2007.00954.x . 17521329 10.1111/j.1462-5822.2007.00954.x

[53] Celli J, Olivier M, Finlay BB. Enteropathogenic Escherichia coli mediates antiphagocytosis through the inhibition of PI 3-kinase-dependent pathways. EMBO J. 2001;20(6):1245–58. doi: 10.1093/emboj/20.6.1245 ; PubMed Central PMCID: PMCPMC145521. 11250891 10.1093/emboj/20.6.1245 PMC145521

[54] Quitard S, Dean P, Maresca M, Kenny B. The enteropathogenic Escherichia coli EspF effector molecule inhibits PI-3 kinase-mediated uptake independently of mitochondrial targeting. Cell Microbiol. 2006;8(6):972–81. doi: 10.1111/j.1462-5822.2005.00680.x . 16681838 10.1111/j.1462-5822.2005.00680.x

[55] Marches O, Covarelli V, Dahan S, Cougoule C, Bhatta P, Frankel G, et al EspJ of enteropathogenic and enterohaemorrhagic Escherichia coli inhibits opsono-phagocytosis. Cell Microbiol. 2008;10(5):1104–15. doi: 10.1111/j.1462-5822.2007.01112.x ; PubMed Central PMCID: PMCPMC2344115. 18201246 10.1111/j.1462-5822.2007.01112.x PMC2344115

[56] Tahoun A, Siszler G, Spears K, McAteer S, Tree J, Paxton E, et al Comparative analysis of EspF variants in inhibition of Escherichia coli phagocytosis by macrophages and inhibition of E. coli translocation through human- and bovine-derived M cells. Infect Immun. 2011;79(11):4716–29. doi: 10.1128/IAI.00023-11 ; PubMed Central PMCID: PMCPMC3257939. 21875965 10.1128/IAI.00023-11 PMC3257939

[57] Iizumi Y, Sagara H, Kabe Y, Azuma M, Kume K, Ogawa M, et al The enteropathogenic E. coli effector EspB facilitates microvillus effacing and antiphagocytosis by inhibiting myosin function. Cell Host Microbe. 2007;2(6):383–92. doi: 10.1016/j.chom.2007.09.012 . 18078690 10.1016/j.chom.2007.09.012

[58] Dong N, Liu L, Shao F. A bacterial effector targets host DH-PH domain RhoGEFs and antagonizes macrophage phagocytosis. EMBO J. 2010;29(8):1363–76. doi: 10.1038/emboj.2010.33 ; PubMed Central PMCID: PMCPMC2868573. 20300064 10.1038/emboj.2010.33 PMC2868573

[59] Goldberg MB, Theriot JA. Shigella flexneri surface protein IcsA is sufficient to direct actin-based motility. Proc Natl Acad Sci U S A. 1995;92(14):6572–6. ; PubMed Central PMCID: PMCPMC41560. 7604035 10.1073/pnas.92.14.6572 PMC41560

[60] Dabiri GA, Sanger JM, Portnoy DA, Southwick FS. Listeria monocytogenes moves rapidly through the host-cell cytoplasm by inducing directional actin assembly. Proc Natl Acad Sci U S A. 1990;87(16):6068–72. ; PubMed Central PMCID: PMCPMC54473. 2117270 10.1073/pnas.87.16.6068 PMC54473

[61] Kuehl CJ, Dragoi AM, Agaisse H. The Shigella flexneri type 3 secretion system is required for tyrosine kinase-dependent protrusion resolution, and vacuole escape during bacterial dissemination. PLoS One. 2014;9(11):e112738 doi: 10.1371/journal.pone.0112738 ; PubMed Central PMCID: PMCPMC4236203. 25405985 10.1371/journal.pone.0112738 PMC4236203

[62] Fattouh R, Kwon H, Czuczman MA, Copeland JW, Pelletier L, Quinlan ME, et al The diaphanous-related formins promote protrusion formation and cell-to-cell spread of Listeria monocytogenes. J Infect Dis. 2015;211(7):1185–95. doi: 10.1093/infdis/jiu546 ; PubMed Central PMCID: PMCPMC4432431. 25281757 10.1093/infdis/jiu546 PMC4432431

[63] Campellone KG, Siripala AD, Leong JM, Welch MD. Membrane-deforming proteins play distinct roles in actin pedestal biogenesis by enterohemorrhagic Escherichia coli. J Biol Chem. 2012;287(24):20613–24. doi: 10.1074/jbc.M112.363473 ; PubMed Central PMCID: PMCPMC3370245. 22544751 10.1074/jbc.M112.363473 PMC3370245

[64] Garmendia J, Carlier MF, Egile C, Didry D, Frankel G. Characterization of TccP-mediated N-WASP activation during enterohaemorrhagic Escherichia coli infection. Cell Microbiol. 2006;8(9):1444–55. doi: 10.1111/j.1462-5822.2006.00723.x . 16922863 10.1111/j.1462-5822.2006.00723.x

[65] Rohatgi R, Nollau P, Ho HY, Kirschner MW, Mayer BJ. Nck and phosphatidylinositol 4,5-bisphosphate synergistically activate actin polymerization through the N-WASP-Arp2/3 pathway. J Biol Chem. 2001;276(28):26448–52. doi: 10.1074/jbc.M103856200 . 11340081 10.1074/jbc.M103856200

[66] Anton IM, Lu W, Mayer BJ, Ramesh N, Geha RS. The Wiskott-Aldrich syndrome protein-interacting protein (WIP) binds to the adaptor protein Nck. J Biol Chem. 1998;273(33):20992–5. . 9694849 10.1074/jbc.273.33.20992

[67] Hyman AA, Weber CA, Julicher F. Liquid-liquid phase separation in biology. Annu Rev Cell Dev Biol. 2014;30:39–58. doi: 10.1146/annurev-cellbio-100913-013325 . 25288112 10.1146/annurev-cellbio-100913-013325

[68] Li P, Banjade S, Cheng HC, Kim S, Chen B, Guo L, et al Phase transitions in the assembly of multivalent signalling proteins. Nature. 2012;483(7389):336–40. doi: 10.1038/nature10879 ; PubMed Central PMCID: PMCPMC3343696. 22398450 10.1038/nature10879 PMC3343696

[69] Banjade S, Rosen MK. Phase transitions of multivalent proteins can promote clustering of membrane receptors. Elife. 2014;3 doi: 10.7554/eLife.04123 ; PubMed Central PMCID: PMCPMC4238058. 25321392 10.7554/eLife.04123 PMC4238058

[70] Jerse AE, Yu J, Tall BD, Kaper JB. A genetic locus of enteropathogenic Escherichia coli necessary for the production of attaching and effacing lesions on tissue culture cells. Proc Natl Acad Sci U S A. 1990;87(20):7839–43. ; PubMed Central PMCID: PMCPMC54845. 2172966 10.1073/pnas.87.20.7839 PMC54845

[71] Murphy KC, Campellone KG. Lambda Red-mediated recombinogenic engineering of enterohemorrhagic and enteropathogenic E. coli. BMC Mol Biol. 2003;4:11 doi: 10.1186/1471-2199-4-11 ; PubMed Central PMCID: PMCPMC317293. 14672541 10.1186/1471-2199-4-11 PMC317293

[72] Fortinea N, Trieu-Cuot P, Gaillot O, Pellegrini E, Berche P, Gaillard JL. Optimization of green fluorescent protein expression vectors for in vitro and in vivo detection of Listeria monocytogenes. Res Microbiol. 2000;151(5):353–60. . 10919515 10.1016/s0923-2508(00)00158-3

[73] Park EK, Jung HS, Yang HI, Yoo MC, Kim C, Kim KS. Optimized THP-1 differentiation is required for the detection of responses to weak stimuli. Inflamm Res. 2007;56(1):45–50. doi: 10.1007/s00011-007-6115-5 . 17334670 10.1007/s00011-007-6115-5

[74] Campellone KG, Webb NJ, Znameroski EA, Welch MD. WHAMM is an Arp2/3 complex activator that binds microtubules and functions in ER to Golgi transport. Cell. 2008;134(1):148–61. doi: 10.1016/j.cell.2008.05.032 ; PubMed Central PMCID: PMCPMC2556884. 18614018 10.1016/j.cell.2008.05.032 PMC2556884

[75] Peterson MD, Mooseker MS. Characterization of the enterocyte-like brush border cytoskeleton of the C2BBe clones of the human intestinal cell line, Caco-2. J Cell Sci. 1992;102 (Pt 3):581–600. . 1506435 10.1242/jcs.102.3.581

[76] Hetrick B, Han MS, Helgeson LA, Nolen BJ. Small molecules CK-666 and CK-869 inhibit actin-related protein 2/3 complex by blocking an activating conformational change. Chem Biol. 2013;20(5):701–12. doi: 10.1016/j.chembiol.2013.03.019 ; PubMed Central PMCID: PMCPMC3684959. 23623350 10.1016/j.chembiol.2013.03.019 PMC3684959

[77] Burgess DR. Reactivation of intestinal epithelial cell brush border motility: ATP-dependent contraction via a terminal web contractile ring. J Cell Biol. 1982;95(3):853–63. ; PubMed Central PMCID: PMCPMC2112935. 7153249 10.1083/jcb.95.3.853 PMC2112935

