# Supplementary figures and images for: Extracellular motility and cell-to-cell transmission of enterohemorrhagic E. coli is driven by EspFU-mediated actin assembly
Source: PLoS Pathog. 2017 Aug 3;13(8):e1006501. doi: 10.1371/journal.ppat.1006501 (PMC5557606; doi:10.1371/journal.ppat.1006501)

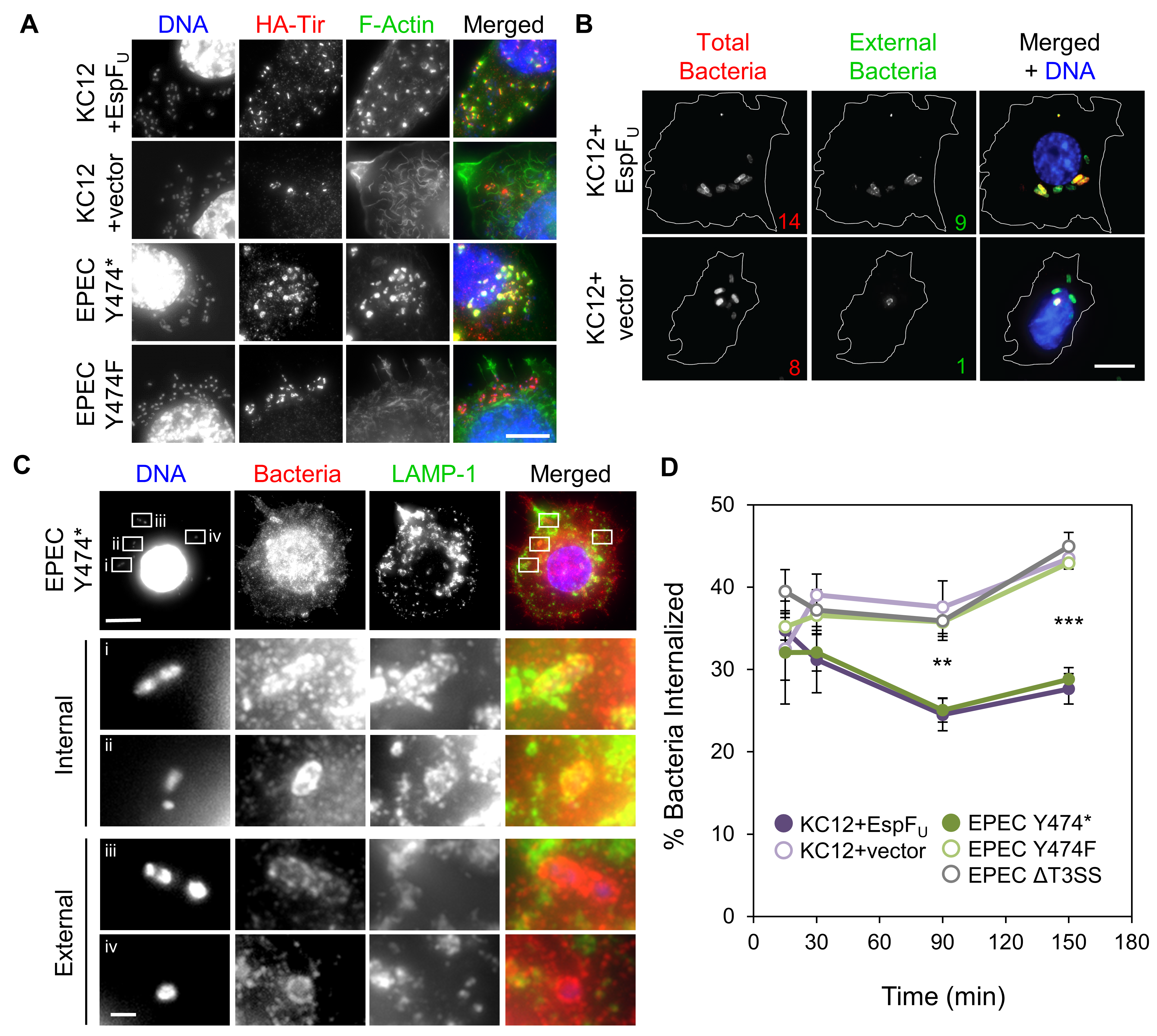

Supplement: S1 Fig — (A) Activated THP-1 macrophages were infected for 3.5 h, fixed, and stained to visualize DNA, HA-Tir, and F-actin. Scale bar, 10 μm. (B) Activated THP-1 cells infected for 90 min were differentially stained to determine the total number of cell-associated bacteria (red), and the number of external bacteria (green). Scale bar, 10 μm. (C) Activated THP-1 cells infected for 90 min with EPEC were fixed and stained for DNA, bacteria, and LAMP-1. Scale bar, 10 μm; inset scale bar, 1 μm. (D) The % of internalized bacteria was quantified at the depicted times. Each data point represents the mean (+/- SE) calculated from 4–6 coverslips with 200–300 total cells spanning at least 3 experiments. **p<0.01, *** p<0.001 (ANOVA, Tukey post-hoc tests). (TIF) [file ppat.1006501.s001.tif]

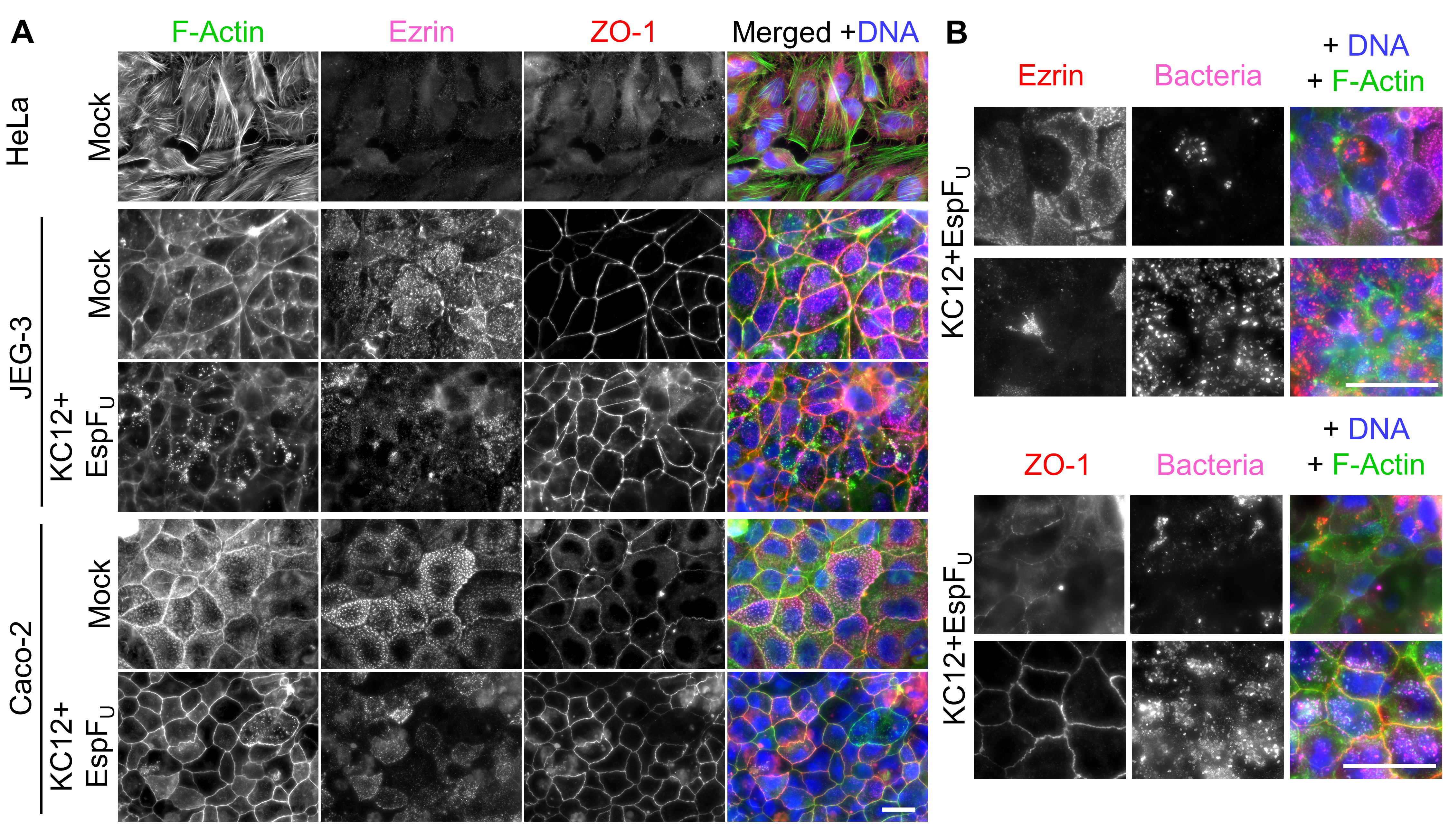

Supplement: S2 Fig — (A) HeLa, JEG-3, or Caco-2 cells were left uninfected or infected with KC12+EspFU for 6 h. Cells were fixed and stained with phalloidin to detect F-actin, Ezrin antibodies to stain microvilli, ZO-1 antibodies to visualize tight junctions, and DAPI to label DNA. Scale bar, 25 μm. (B) Polarized Caco-2 monolayers were infected with KC12+EspFU for 6 h, fixed, and stained to detect Ezrin (top) or ZO-1 (bottom), in addition to bacterial LPS, DNA, and F-actin. Areas of low (first and third rows) and high (second and fourth rows) bacterial burdens were imaged from the same coverslip for each staining condition. Scale bar, 50 μm. (TIF) [file ppat.1006501.s002.tif]

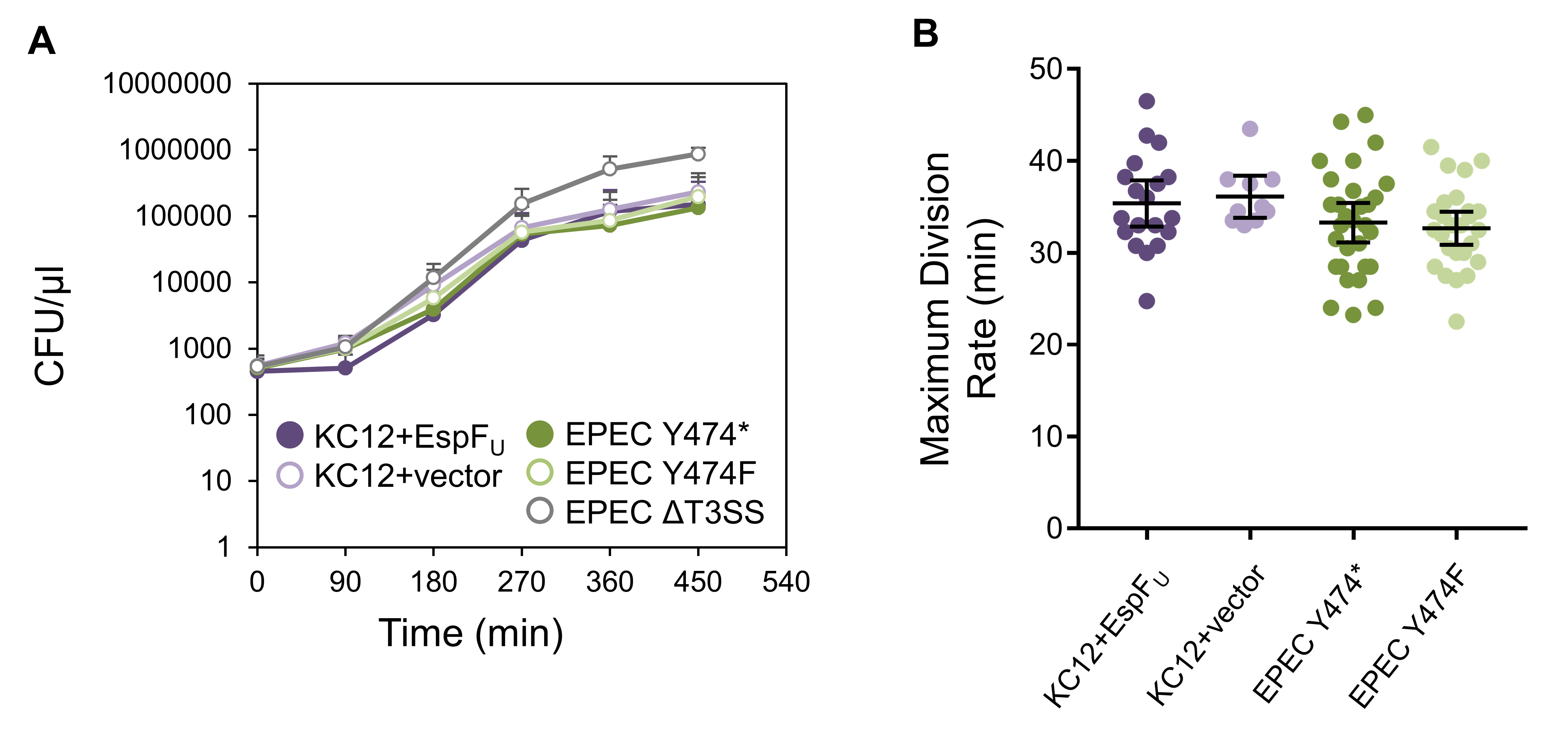

Supplement: S3 Fig — (A) Bacteria grown in infection media were diluted and plated every 90 min to determine the number of Colony Forming Units (CFUs). Each data point represents the mean number of CFUs (+SD) from 4 experiments. (B) JEG-3 cells were infected for 6 h with the indicated strains and imaged live. Individual bacteria were tracked over time to determine the amount of time between consecutive divisions and calculate the maximum division rate. Each point represents a single bacterium, with the mean (+/- SD) indicated in black. (TIF) [file ppat.1006501.s003.tif]

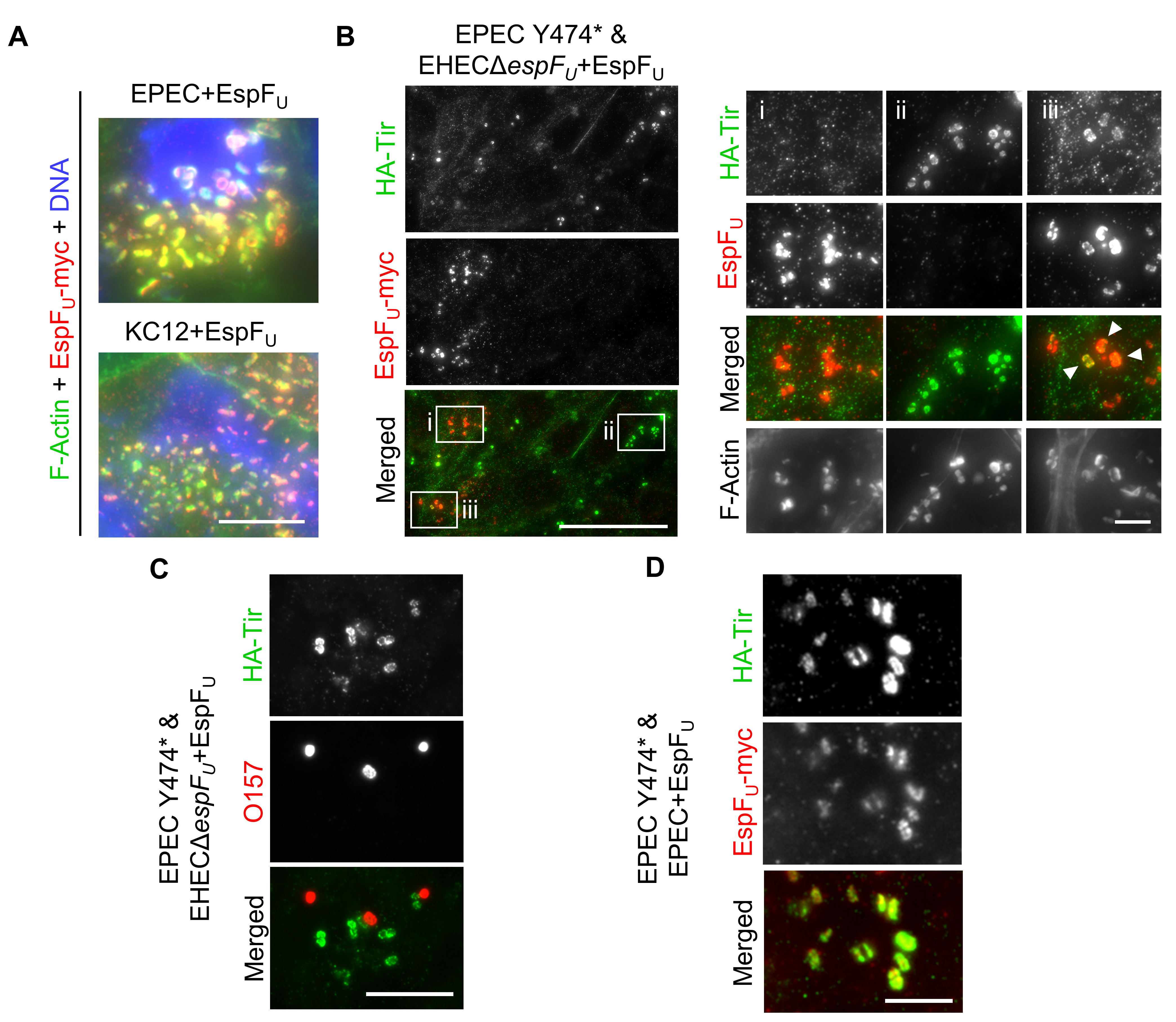

Supplement: S4 Fig — (A) Polarized Caco-2 monolayers were infected with EPEC+EspFU or KC12+EspFU, fixed and stained for EspFU-myc, F-actin, and DNA. Scale bar, 10 μm. (B) JEG-3 monolayers were co-infected for 6 h with equal amounts of EPEC Y474* and EHECΔespFU+EspFU, fixed, and stained for HA-Tir (which is only tagged in EPEC), EspFU-myc (which is only expressed by EHEC), F-actin, and DNA. Areas of isolated EHEC bacteria (i), isolated EPEC bacteria (ii), or mixed infection (iii) are shown in insets. Colocalization between HA-Tir and EspFU-myc is highlighted with arrowheads, indicating that bacteria can share pedestal effectors during co-infection. Scale bars, 50 μm, inset 5 μm. (C) JEG-3 monolayers were co-infected and fixed as in (B), but stained for EHEC O157 in addition to HA-Tir. Colocalization between HA-Tir and O157 was not observed, suggesting that Tir is not effectively transferred from EPEC to EHEC. Scale bars, 50 μm, inset 12.5 μm. (D) JEG-3 monolayers were co-infected with EPEC Y474* and EPEC+EspFU, fixed, and stained for HA-Tir (which is only tagged in EPEC Y474*) and EspFU-myc (which is only expressed in EPEC+EspFU). Colocalization indicates that EPEC strains can efficiently share HA-Tir and EspFU. Scale bar, 5 μm. (TIF) [file ppat.1006501.s004.tif]

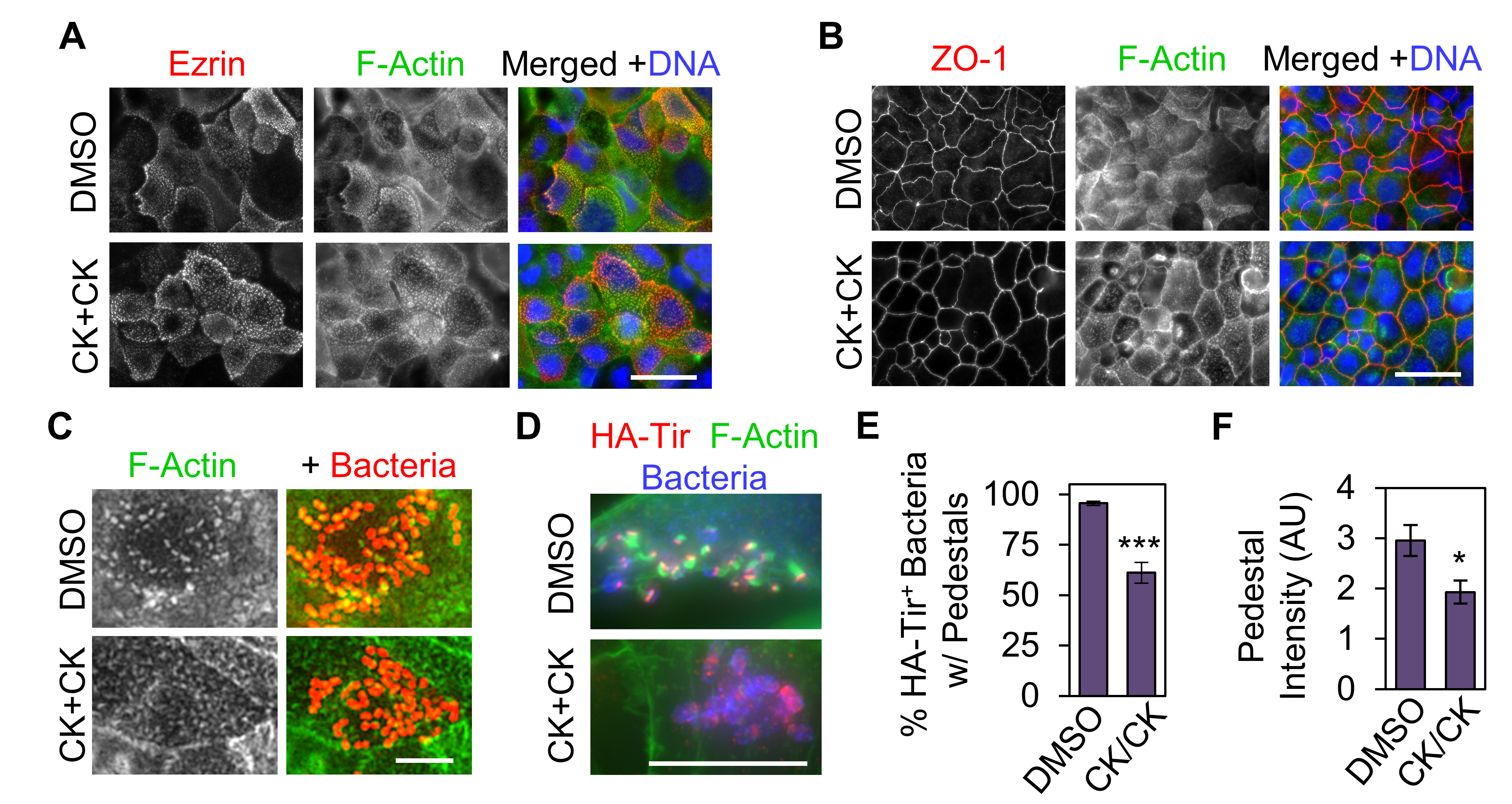

Supplement: S5 Fig — (A-B) Polarized Caco-2 monolayers were treated with either DMSO or CK666+CK869 for 6 h, fixed, and stained for DNA, F-actin, and either Ezrin or ZO-1. Scale bar, 50 μm. (C) Polarized Caco-2 monolayers were pretreated for 15 min with DMSO or CK666+CK869, then infected for 6 h with KC12+EspFU in the presence of either DMSO or inhibitors. Cells were fixed and stained for F-actin and bacteria. Scale bar, 10 μm. (D) NIH3T3 cells were infected with KC12+EspFU and treated with DMSO or CK666+CK869 for 4 h, fixed, and stained for HA-Tir, F-actin, and bacteria. Scale bar, 10 μm. (E) The % of HA-Tir positive foci that were associated with actin pedestals from experiments in (D) was calculated. Each bar represents the mean (+/- SE) calculated from 15 cells, each harboring up to 50 bacteria. (F) The relative intensity of pedestals associated with HA-Tir foci was calculated and normalized to an adjacent pedestal-free area of the cell that was set to 1. Each bar represents the mean (+/- SE) calculated from 9 cells harboring up to 40 pedestals per cell. *p<0.05, ***p<0.001 (unpaired t tests). (TIF) [file ppat.1006501.s005.tif]
