# Supplementary material for: Extracellular motility and cell-to-cell transmission of enterohemorrhagic E. coli is driven by EspFU-mediated actin assembly
Source: PLoS Pathog. 2017 Aug 3;13(8):e1006501. doi: 10.1371/journal.ppat.1006501 (PMC5557606; doi:10.1371/journal.ppat.1006501)
Supplement: S1 Table — (PDF) [file ppat.1006501.s006.pdf]

Supporting Information

Supplementary Table 1: Strains used in this study.

Name	Strain + Plasmid	Description	Reference
WT EPEC	JPN15/pMAR7	Amp^r derivative of EPEC E2348/69 (O127:H6)	(70)
EPEC Y474*	KC14+pKC17	EPECΔ*tir*+pHA-tir(WT)	(22)
EPEC Y474F	KC14+pKC142	EPECΔ*tir*+pHA-tir(Y474F)	(22)
EPECΔ*tir*	KC14	EPECΔ*tir*	(22)
EPECΔT3SS	KC30	EPECΔT3SS	(71)
EPEC+GFP	WT EPEC+pAT113	EPEC+pEGFP	This study, (72)
EPEC+$EspF_U$	WT EPEC+pKC471	EPEC+p$EspF_U$-Myc	This study
EPEC+vector	WT EPEC+pKC469	EPEC+pMyc	This study
WT EHEC	TUV93-0	Stx^- derivative of EDL933 (O157:H7)	(22)
EHECΔ$espF_U$+$EspF_U$	KC44+pKC471	EHECΔ$espF_U$+p$EspF_U$-Myc	(27)
EHECΔ$espF_U$+vector	KC44+pKC469	EHECΔ$espF_U$+pMyc	(27)
KC12	KC12	EPECΔ*tir-cesT-eae*::EHEC-*HA-tir-cesT-eae*	(22)
KC12+$EspF_U$	KC12+pKC471	KC12+p$EspF_U$-Myc	(27)
KC12+vector	KC12+pKC469	KC12+pMyc	(27)
KC12Δ*tir*	KC26	KC12Δ*tir*	(22)
KC12Δ*tir*+$EspF_U$	KC26+pKC471	KC12Δ*tir*+p$EspF_U$-Myc	This study
KC12Δ*tir*+vector	KC26+pKC469	KC12Δ*tir*+pMyc	This study
