# Supplementary material for: Extracellular motility and cell-to-cell transmission of enterohemorrhagic E. coli is driven by EspFU-mediated actin assembly
Source: PLoS Pathog. 2017 Aug 3;13(8):e1006501. doi: 10.1371/journal.ppat.1006501 (PMC5557606; doi:10.1371/journal.ppat.1006501)
Supplement: S2 Table — (PDF) [file ppat.1006501.s007.pdf]

Supplementary Table 2: Antibodies and molecular probes used in this study.

Target	Antibody/Probe	Animal	Concentration	Fixation	Company
Primary Antibodies:					
LPS	anti-Lipopolysaccharide	Mouse	2 µg/ml	PFA	Abcam
Bacteria	Rabbit serum	Rabbit	1%	PFA	Covance
O157	O157 Antiserum	Rabbit	0.01%	PFA	Difco
Tight junctions	anti-TJP1 (ZO-1)	Mouse	1 µg/ml	PFA	Life Technologies
Microvilli	anti-Ezrin	Rabbit	1:500	PFA	Cell Signaling Technologies
HA-Tir	anti-HA.11	Mouse	1 µg/ml	PFA	Covance
HA-Tir	anti-HA.11	Rabbit	2 µg/ml	PFA	Covance
$EspF_U$-Myc	anti-Myc	Mouse	2 µg/ml	PFA	Sigma
Early lysosomes	anti-LAMP-1	Mouse	2 µg/ml	Methanol	Santa Cruz Biotechnologies
Secondary Antibodies:					
Mouse IgG	Alexa555,568,488 anti-mouse	Goat	4 µg/ml	PFA or Methanol	Life Technologies
Rabbit IgG	Alexa555,568,488 anti-rabbit	Goat	4 µg/ml	PFA or Methanol	Life Technologies
Other:					
F-actin	Alexa488-Phalloidin		2 U/ml	PFA	Life Technologies
DNA	4′,6-diamidino-2-phenylindole (DAPI)		1 µg/ml	PFA or Methanol	Life Technologies
